# Supplementary material for: Development of an efficient, effective, and economical technology for proteome analysis
Source: Cell Rep Methods. 2024 Jun 11;4(6):100796. doi: 10.1016/j.crmeth.2024.100796 (PMC11228373; doi:10.1016/j.crmeth.2024.100796)
Supplement: Document S1. Figures S1–S4, Table S1, and Methods S1 [file mmc1.pdf]

Cell Reports Methods, Volume *4*

Supplemental information

Development of an efficient, effective,

and economical technology for proteome analysis

Katherine R. Martin, Ha T. Le, Ahmed Abdelgawad, Canyuan Yang, Guotao Lu, Jessica L. Keffer, Xiaohui Zhang, Zhihao Zhuang, Papa Nii Asare-Okai, Clara S. Chan, Mona Batish, and Yanbao Yu

Figure S1. Evaluation of glass bead membrane for proteomics analysis, related to Figure 1.

(A) Representative scanning electron microscopy imaging of the glass bead membrane (30 μm, 100X). (B) Comparison of protein and peptide identifications derived from the two types of GB membranes (9-13 μm, and 30 μm). E. coli samples were tested for this experiment. (C) Comparison of number of quantified proteins in different replicates. (D) Overlapping analyses of protein (upper panel) and peptide (lower panel) hits obtained by triplicate experiments of the three methods, E3filter, FASP, and SP4-GB.

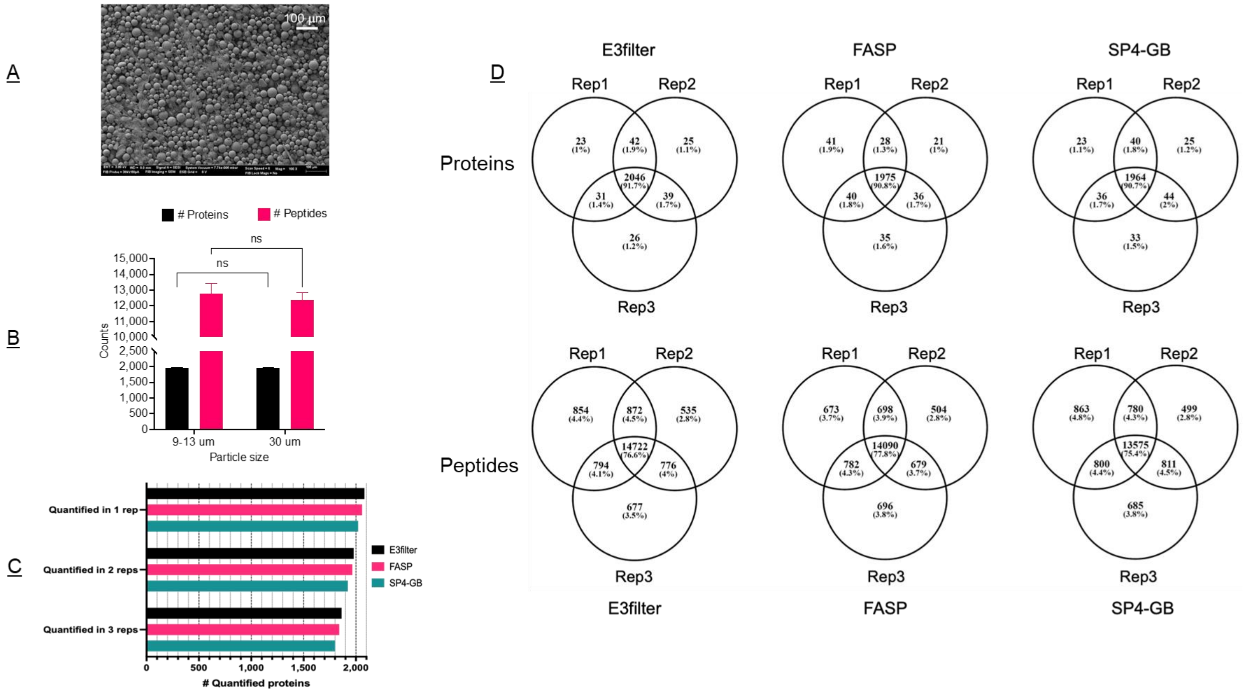

Figure S2. Proteomic comparison of E3tip and FA-SPEED methods, related to Figure 2.

(A) Correlation analysis of triplicate experiments of E3tip and FA-SPEED. Protein (left panel) and peptide (right panel) intensity (LFQ value, log2) of each experiment was plotted. Pearson r values (in blue) are depicted in the plots. (B) Comparison of FA-SPEED procedures with and without neutralization. Overlaps of *E.coli* protein and peptide identifications by the two procedures. The data were from three biological replicates. (C) Unsupervised hierarchical clustering analysis of the two procedures. Color bar indicates the protein intensity (LFQ, log2).

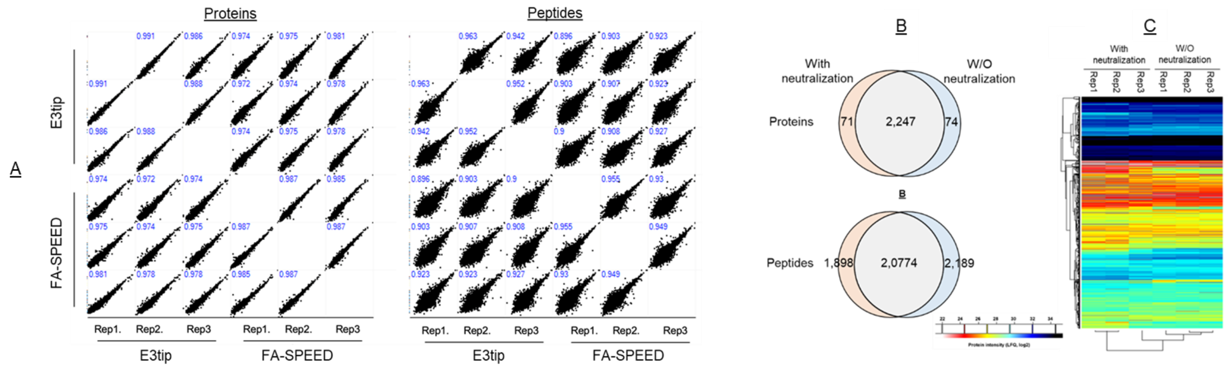

Figure S3. Applying E3tip to AP-MS analysis, related to Figure 5.

(A-B) Quantitative analyses of the two pulldown assays using linearRNA (A) and circRNA (B), respectively. The protein YBX1 was highlighted (red circle) in the plots. The curves indicate Permutation FDR 0.05. (C-H) Extended images of CircNFIX and YBX1 interaction. (C) DIC; (D) DAPI staining; (E) Raw merged z-stacks of Texas red (for probe set exclusively binding to linear RNA); (F) Raw merged z-stacks of Cy5 (probe set binding to exon found in both linear and circular RNA); (G) Raw merged z-stacks of Alexa 488 (anti-YBX1 antibody); (H) Merged image of the three channels showing colocalization. Full-length linear RNAs are represented as yellow, circular RNA as green, and YBX1 protein is represented in blue.

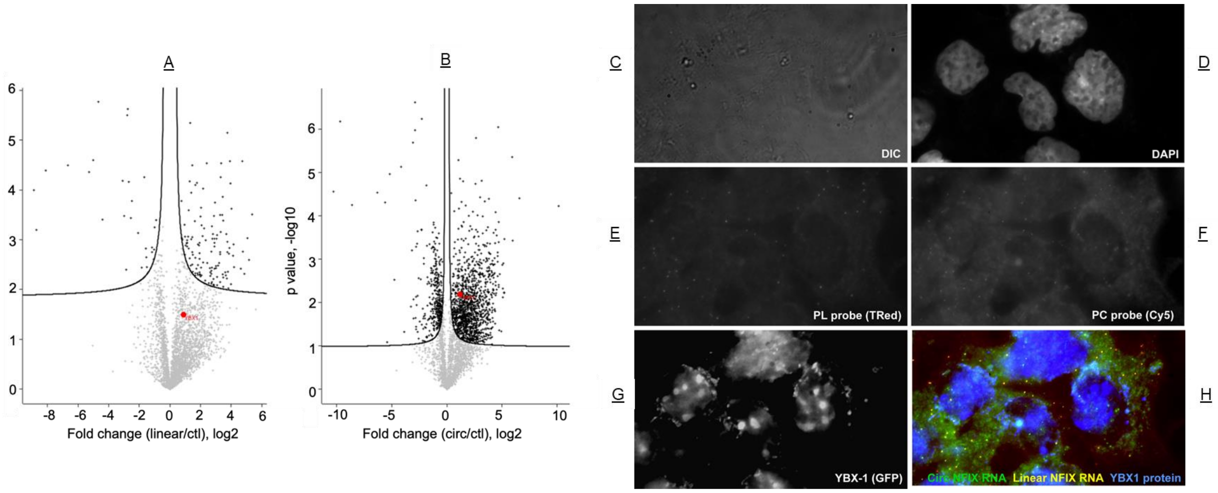

Figure S4. Evaluation of on-filter in-cell digestion, related to Figure 6.

(A) LCMS base peak profile of two representative digestions. Upper panel, on-filter in-cell digestion of ES-1 cells. Lower panel, SDS lysis of ES-1 cells and PES membrane followed by STrap digestion. (B-D) Proteomic comparison of “OFIC-E4” and “TFA-E3” digestion methods using *Leptothrix cholodnii* (SP-6) cells. The numbers of protein and peptide identifications, and the percentages of mis-cleavages were plotted. In this experiment, the SP6 cell pellets were either lysed with TFA followed by E3filter digestion (post protein precipitation), or on-filter in-cell digested with E4filter. Four biological replicates were included for each digestion experiment.

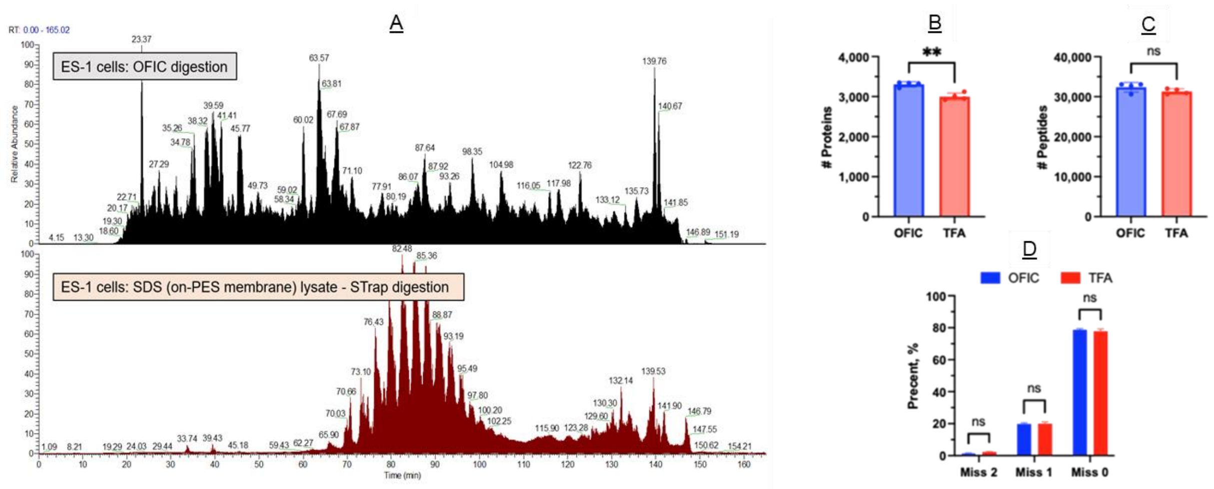

Table S1. Sequences of the probes used in this study, related to Figure 5.

probe name	Sequence of the probes	probe name	Sequence of the probes
PC probe set	tcgatgaacgggtggaactc	PL probe set	tcttatcagaggaaccagga
	Gcggacgtgaggcagcagtg		caagggctctctgagaactg
	Tgaaccaggtgtaggagaaa		tacttgacagagtccatgtc
	Ttgcgcttccgcgcctgcag		agaattgtgctggttgcttt
	Cttttcatgcttcttgaagt		attctgggacacgcaactag
	Gctcctcgtccttcgacatc		tcgagagcattccgactttt
	Cagcagctcgtccttcaccg		aactgaaagtcggcgagcag
	Gcttgatctcgggcttctcg		aaagtcggggggcacagaaa
	Agcagccgggatgcccactt		ccacccctaaataaagagta
	Gatgtccttgcgcagcttgg		tcccattccaacagcaaaag
	Agtcctcgcggaactcgggc		gttgctcaaatggaggagga
	Ttgcccgtgatggtcagcac		gtgtgttaccaaattgttcc
	Tgcccttctggtcggggttg		acaaggactcaagaaggggc
	Aggcagtcaatccgccggat		ttctctcagaggatcctgac
	Ccacaccttgtcagcctggc		ctcacaacaccacttgggaa
	Tcaccatgaccaggtccagc		aactctctgatgcattgcac
	Agggggatccccttaaacaa		cctcgtcaacgcaggaaaac
	Ccgctccccatcagtacttt		tcccaccgaaacagaacgaa
	Agcactgaggcgacttgtag		taatgtaagaagcaccaggg
	Tggacgcacaggccggggtt		tgcgtattcctaacaagtgc
	Tgtgactccaatgtgatgtg		ccgttccgttaaactcaaca
	Gataaagatccagttctttg		gtttgttcgttggcattgac
	Ggagtgtggacaaagtaagc		tggcgtctggctcaaagaag
			tgataatgctggtgagggtc
			ggggaggaaactaccaactt
			tcggggataggatgagagat
			aaatcgacctgtcagcgtgg
			tgtataaggcagtcgacagg
			gggtgagaacaaggcactag
			gggacgaaagttctcgtgac
			aacttgtttgacgggacggg
			ctcgtatatactgcgtttct
			agcacaccaaatccattagt
			tgtatctcagtgcagagacg
			agtaaactacccttgtttct

Supplemental Methods S1. Detailed protocols of E3technology and E4technolog, related to STAR Methods.

E3technology for proteomics sample preparation

Protein Digestion of Cell Lysate

Before starting

- Collect samples such as cell pellets, tissues, body fluids, etc.
- Lysis samples with buffers on your own choice.
- Calculate protein concentration and aliquot certain amount for proteomics.
- Estimated loading capacity: **E3tip**, <20 μg; **E3filter**, 10-100 μg; **E3cartridge**, 50-500 μg; **E3plate**, 20-200 μg.
- Ready-to-go E3 products are available.

E3filter procedure

(Below is a representative procedure for E3filter. Please adjust buffer volume and centrifugation speed according to your filter type)

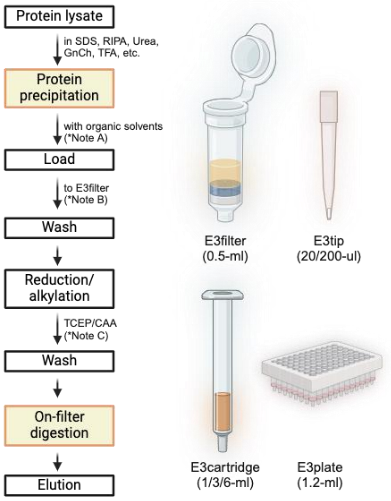

1. **Protein precipitation**
   Depending on sample volume, add 4x of 80% acetonitrile (ACN) to protein lysate to induce protein precipitation. **Note A**: For TFA lysate, acetone is recommended; for Guanidine hydrochloride lysate, ethanol is recommended for protein precipitation.
2. **Sample loading**
   Transfer protein precipitate to E3filters, centrifuge at 400 x g for 1-2 min; discard flow through. Centrifugation speed may be increased (up to 7,000 x g) if more proteins are loaded. **Note B**: when handling low-input samples (e.g., <10 μg), the protein lysate may be added directly to E3filter followed by protein precipitation with organics.
3. **Wash**
   Add 200 μl 80% ACN, centrifuge at 400 x g for 1-2 min, and discard flow through. Repeat this step 2-3 times.
4. **Reduction and alkylation**
   Add 100 μl 50 mM triethylammonium bicarbonate (TEAB), 10 mM Tris(2-carboxyethyl)phosphine (TCEP), and 40mM chloroacetamide (CAA), incubate at 45°C for 5 min with gentle shaking. Spin and discard flow through.
   Note C: this step may be skipped if it were performed ahead (e.g., during cell lysis step).
5. **Wash**
   Same as Step 3.
6. **Digestion**
   Transfer E3filters to clean collection tubes, add 100-200 μl 50 mM TEAB, and desired enzyme (Trypsin or Trypsin/Lys-C mix) at 1:50 ratio. Incubate E3filters at 37°C for 16-18 hours with gentle shaking (e.g., 300-500 rpm).
7. **Elution**
   Centrifuge E3filters at 400 x g for 1-2 min, transfer elutes to new collection tubes. Perform two additional elution steps with 0.1% formic acid in water, and 50% acetonitrile/0.1% formic acid in water, respectively. Pool the elution, dry, and proceed to desalting, or store at -80°C until further use.

Martin et al., Development of an efficient, effective, and economical technology for proteome analysis,
Cell Reports Methods (2024), https://doi.org/10.1016/j.crmeth.2024.100796

E4technology for proteomic sample preparation

Protein Digestion of Intact Cells

Before starting

- Collect fresh cell pellets or tissue slices, rinse with cold PBS to remove excess culturing media or blood.
- Count cells and aliquot certain amount for proteomics.
- Estimated capacity: **E4tip**, ≤ 50,000 cells; **E4filter**, 10,000-100,000 cells; **E4cartridge**, 100,000-1,000,000 cells; **E4plate**, 50,000-500,000 cells.
- Ready-to-go E4 products are available.

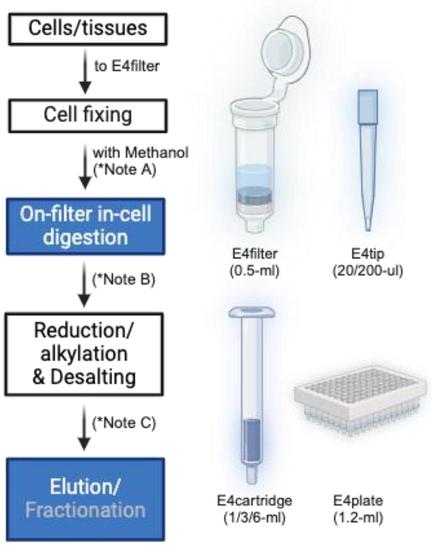

E4 procedure

(Below is a representative procedure for E4filter; please adjust buffer volume and centrifugation speed according to your filter type)

1. **Cell loading and fixing**
   Transfer certain amount of cells to E4filter, add 200 μl of 100% methanol, and mix cells by gentle pipetting. Incubate on ice or at 4°C for 0.5-2.0 hours. Centrifuge at 1,500 x g for 1-2 min, and discard flow through. Here, centrifugation speed may go up to 7,000 x g if more cells are loaded.
   Note A: The flow through (extraction) here may be collected for ***metabolomics*** analysis.
2. **Digestion**
   Transfer E4filters to clean collection tubes, add 100-200 μl 50 mM TEAB, and desired enzyme (Trypsin or Trypsin/Lys-C mix) at 1:50 ratio. Incubate at 37°C for 16-18 hours with gentle shaking (e.g., 300-500 rpm). For E4tips, no caps are required during incubation. Only minor liquid loss from evaporation can be seen.
3. **Reduction/alkylation**
   After digestion, before doing any centrifugation, add 10 mM (final concentration) Tris(2-carboxyethyl)phosphine (TCEP) and 40mM (final concentration) chloroacetamide (CAA), incubate at 45°C for 5 min with gentle shaking.
4. **Acidification and desalting**
   Add formic acid to final concentration of 1%, centrifuge at 1,500 x g for 1-2 min, and discard flow through. Add 200 μl 0.5% acetic acid in water, spin and discard flow through. The desalting step may be repeated one more time.
   Note B: If E4tips are used, the tips are now desalted, and are ready for elution (**Step 5**), in-tip high-pH fractionation, or direct LCMS acquisition (e.g., if EvoSep LC is accessible).
5. **Elution (or Fractionation)**
   Transfer E4filters to clean collection tubes. Do two sequential elution by adding 200 μl 60% acetonitrile/0.5% acetic acid in water (elution I), and 80% acetonitrile/0.5% acetic acid in water (elution II), respectively. Centrifuge at 1,500 x g for 1-2 min, pool the elution, dry, store at -80°C until LCMS analysis.
   Note C: If in-depth proteome coverage is desired, high pH fractionation may be carried out at this step (by doing sequential elution).

Martin et al., Development of an efficient, effective, and economical technology for proteome analysis,
Cell Reports Methods (2024), https://doi.org/10.1016/j.crmeth.2024.100796
