# Supplementary material for: Development of an efficient, effective, and economical technology for proteome analysis
Source: Cell Rep Methods. 2024 Jun 11;4(6):100796. doi: 10.1016/j.crmeth.2024.100796 (PMC11228373; doi:10.1016/j.crmeth.2024.100796)
Supplement: Document S2. Article plus supplemental information [file mmc2.pdf]

Cell Reports
Methods

Article

Development of an efficient, effective, and economical technology for proteome analysis

Graphical abstract

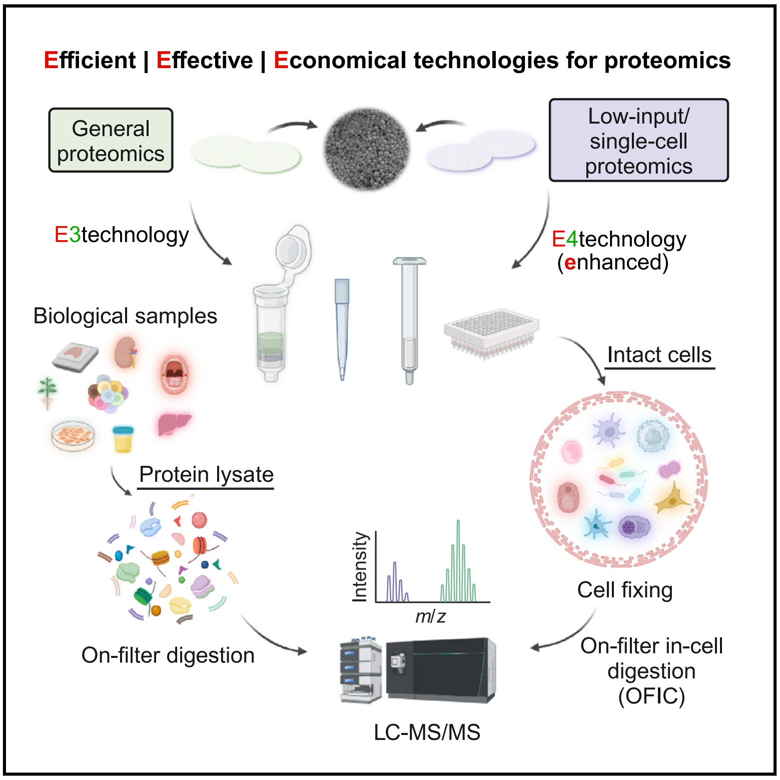

Authors

Katherine R. Martin, Ha T. Le, Ahmed Abdelgawad, ..., Clara S. Chan, Mona Batish, Yanbao Yu

Correspondence

batish@udel.edu (M.B.), yybyu@udel.edu (Y.Y.)

In brief

Martin et al. present an attractive alternative to proteomics sample preparation, named E3technology, which is simple, rapid, reliable, affordable and has versatile formats. An enhanced single-vessel approach, named E4technology, can process intact cells directly with minimal sample loss, facilitating low-input/low-cell proteomics analysis.

Highlights

- Rapid, robust, and cost-effective alternative to proteomics sample preparation
- Versatile filter devices can meet a wide range of proteomics analysis needs
- On-filter in-cell digestion facilitates low-input proteomics
- Ready-to-go E3 and E4 filter devices are available

Martin et al., 2024, Cell Reports Methods *4*, 100796
June 17, 2024 © 2024 The Authors. Published by Elsevier Inc.
https://doi.org/10.1016/j.crmeth.2024.100796

Cell Reports Methods

CellPress OPEN ACCESS

Article

Development of an efficient, effective, and economical technology for proteome analysis

Katherine R. Martin,[1] Ha T. Le,[1,7] Ahmed Abdelgawad,[2,3,7] Canyuan Yang,[1,7] Guotao Lu,[4] Jessica L. Keffer,[5] Xiaohui Zhang,[4] Zhihao Zhuang,[1] Papa Nii Asare-Okai,[1] Clara S. Chan,[5,6] Mona Batish,[2,3,*] and Yanbao Yu[1,8,*]

[1]Department of Chemistry and Biochemistry, University of Delaware, Newark, DE 19716, USA
[2]Department of Biological Sciences, University of Delaware, Newark, DE 19716, USA
[3]Department of Medical and Molecular Sciences, University of Delaware, Newark, DE 19716, USA
[4]CDS Analytical, LLC, Oxford, PA 19363, USA
[5]Department of Earth Sciences, University of Delaware, Newark, DE 19716, USA
[6]School of Marine Science and Policy, University of Delaware, Newark, DE 19716, USA
[7]These authors contributed equally
[8]Lead contact
*Correspondence: batish@udel.edu (M.B.), yybyu@udel.edu (Y.Y.)
https://doi.org/10.1016/j.crmeth.2024.100796

MOTIVATION Conventional proteomics sample processing methods often have high technical barriers to broad biomedical scientists, leading to difficulties for quick adoption and standardization. Existing protocols are also typically associated with costly reagents and accessories, making them less feasible for resource-limited settings as well as for clinical proteomics and/or core facilities where large numbers of samples are usually processed. Thus, there is a strong unmet need for an easy-to-use, reliable, and low-cost approach for general proteomics sample preparation.

SUMMARY

We present an efficient, effective, and economical approach, named E3technology, for proteomics sample preparation. By immobilizing silica microparticles into the polytetrafluoroethylene matrix, we develop a robust membrane medium, which could serve as a reliable platform to generate proteomics-friendly samples in a rapid and low-cost fashion. We benchmark its performance using different formats and demonstrate them with a variety of sample types of varied complexity, quantity, and volume. Our data suggest that E3technology provides proteome-wide identification and quantitation performance equivalent or superior to many existing methods. We further propose an enhanced single-vessel approach, named E4technology, which performs on-filter in-cell digestion with minimal sample loss and high sensitivity, enabling low-input and low-cell proteomics. Lastly, we utilized the above technologies to investigate RNA-binding proteins and profile the intact bacterial cell proteome.

INTRODUCTION

The ultimate goal of a typical proteomics analysis is to analyze all the proteins, the so-called proteome, of a biological sample so that the biology- and/or pathology-relevant molecules and marker proteins, especially those in low abundance, can be revealed. Therefore, a major component of a bottom-up/shotgun proteomic experiment is sample preparation, which includes cell lysis and protein extraction, cleanup and digestion, and peptide desalting and/or fractionation.[1] Efficient cell lysis is critical to achieve unbiased protein extraction with a high yield. A variety of cell disruption methods, including chemical (e.g., urea, SDS, trifluoroacetic acid [TFA], etc.) and physical (e.g., sonication, bead beating, homogenization, etc.) based, have been employed by the community.[2,3] For protein digestion, it can occur either in solution, given the proteins are fully denatured and no enzyme-interfering chemicals are present, or on a solid support, such as polyacrylamide gel,[4] membranes,[5–8] magnetic beads,[9] or a hybrid format (e.g., immobilized beads in membranes).[10] Their commercialization and the resulting ready-to-go products have provided great convenience to the community and facilitated wide adoption of the methods.[2,11] Unfortunately, most of these commercial products are costly. For instance, compared to the DNA extraction columns that are commonly used for plasmid DNA preparation in genomic science and are commercially available from over 20 vendors (e.g., Miniprep spin columns), the devices for proteomics sample preparation are not only limited in the market but also are 3–20 times more expensive. The reasons

© 2024 The Authors. Published by Elsevier Inc.
This is an open access article under the CC BY license (http://creativecommons.org/licenses/by/4.0/).

for the high cost are likely related to the restriction of materials, limitations on manufacturing capacity, or simply the limited number of vendors competing in the market. Thus, there is a great need for developing and commercializing new or alternative methods that can have the combined merits of cost effectiveness, efficiency, good tolerance to detergents, and robustness.

Organic solvents have been well known to induce protein precipitation, which can be used to eliminate contaminations and purify proteins.[12,13] In the context of proteomics experiments, the precipitated proteins may be resolubilized followed by in-solution digestion.[11] Alternatively, the proteins can be precipitated directly onto magnetic beads with subsequent on-bead digestion. Such a method has been established as the single-pot, solid-phase-enhanced sample preparation (SP3) technology.[9] However, SP3 suffers from typical concerns related to nearly all free-bead-based processing methods, such as potential sample loss to tube walls and pipette tips,[14] unintentional disruption of protein aggregates,[15] inconsistent aliquoting of bead suspensions or insufficient distribution of beads due to rapid sedimentation,[16] and possible cross-contaminations during automation.[17] In addition, SP3 processing requires pH adjustment, is protein concentration-dependent, and has to meet a certain bead-protein ratio, all of which increase its technical barrier for standardization and quick adoption even by non-expert proteomics scientists. Interestingly, a recent study revealed that on-bead protein aggregation is independent of bead surface chemistry.[15] Another study by Johnston et al. further demonstrated that the solid phase could be omitted entirely, favoring a conventional "precipitation-resolubilization-in solution digestion" approach.[14] Although the authors also investigated protein precipitation onto inert glass beads (GBs), they claimed only marginal advantages over a centrifugation-based, bead-free method.

In the present study, we set out to explore a hybrid version of the existing free-bead and bead-free methods—immobilized beads. Immobilized chromatographic beads in polytetrafluoroethylene (PTFE) meshwork have been widely known as Empore membranes. In particular, the C18-based membrane has been widely employed by the proteomics community in the form of a stop-and-go-extraction tip (StageTip).[18] Membranes immobilized with other types of chromatographic beads such as ion-exchange resins were also explored for protein cleanup, digestion, and fractionation.[10,19–23] Empore membranes hold great advantage over free beads. This is because loose particles are entrapped in the PTFE matrix, making them much easier to work with (e.g., aliquoting, transferring, etc.). In addition, since the Empore membrane can be conveniently packed into individual filter devices or multi-well plates, the membrane-based sample preparation is readily scalable and automatable, with no need for extensive method adjustment or reoptimization.[17] Here, for the first time, we combine the Empore technology with GBs to build an immobilized GB membrane and develop an efficient, effective, and economical approach, named E3technology, for proteomics sample preparation. We benchmark its performance using different formats and a variety of sample types of varied complexity, volume, and quantity. We compare them side by side with several established methods and evaluate the quantitative and qualitative performance of the E3technology. We further developed an enhanced "single-vessel" approach, named E4technology, to process low-input samples. Lastly, we employed the developed technologies to identify RNA-binding proteins (RBPs) and profile the intact bacterial cell proteome.

RESULTS

Initial evaluation of E3technology for global proteomics analysis

Design

We first set out to examine the feasibility of using a GB membrane for proteomics sample preparation. We compared its performance with filter-aided sample preparation (FASP),[6] one of the standard filter-based methods that utilizes molecular weight cutoff membranes to process proteins for mass spectrometric analysis. We also performed a comparison with another digestion method that utilizes free GBs, the so-called SP4-GB method.[14] In this method, the proteins are precipitated onto GBs by acetonitrile followed by on-bead digestion. In addition, we tested two different sizes of the GBs, one was in the 10 μm range and the other was around 30 μm. Initial inspection of the GB membranes revealed that the beads were held in place and embedded in the PTFE meshwork (Figure S1A). A pilot digestion experiment indicated that the two GB membranes provided equivalent identification performances (Figure S1B). The following experiments in this study utilized the 30 μm GB membranes.

Qualitative assessment

We initially explored the *E. coli* proteome to assess the qualitative and quantitative proteomic performance of the GB membrane. Here, we built a prototype E3filter by assembling the membrane into a 0.5 mL filter device as we used previously.[24] From three independent digestion experiments and with an input of about 20 μg *E. coli* lysate and a single-shot liquid chromatography-mass spectrometry (LCMS) run, the E3filter was able to identify, on average, 2,207 proteins and 17,358 peptides, which were consistently higher than the FASP and SP4-GB methods that were also tested here (Figures 1A and 1B). FASP appeared to provide slightly more peptide spectrum matches (PSMs), yet they did not translate to more unique peptides. Over 93% of the protein hits and 80% of the peptide hits derived from either FASP or SP4-GB were also identified by the E3filter (Figures 1D and 1E), suggesting that on-GB membrane digestion is not biased toward any particular proteins or protein groups. Meanwhile, the qualitative reproducibility of the E3filter was high as well. Identification overlaps between the triplicate experiments were >95% for proteins and >85% for peptides, consistent with the performances of the FASP and SP4-GB methods (Figure S1D). We next examined the missed cleavages to assess the efficiency of on-membrane digestion. Around 83% of the peptides derived from the E3filters were completely digested. Although this is slightly lower than the SP4-GB method (88%), it is still better than FASP (81.5%) (Figure 1I). These data imply that the immobilization of GBs to PTFE may have a negligible impact on the efficiency of proteolytic digestion.

Quantitative characterization

We observed excellent quantitative reproducibility of the E3technology (Figure 1H). The Pearson correlation among the triplicate

CellPress
OPEN ACCESS

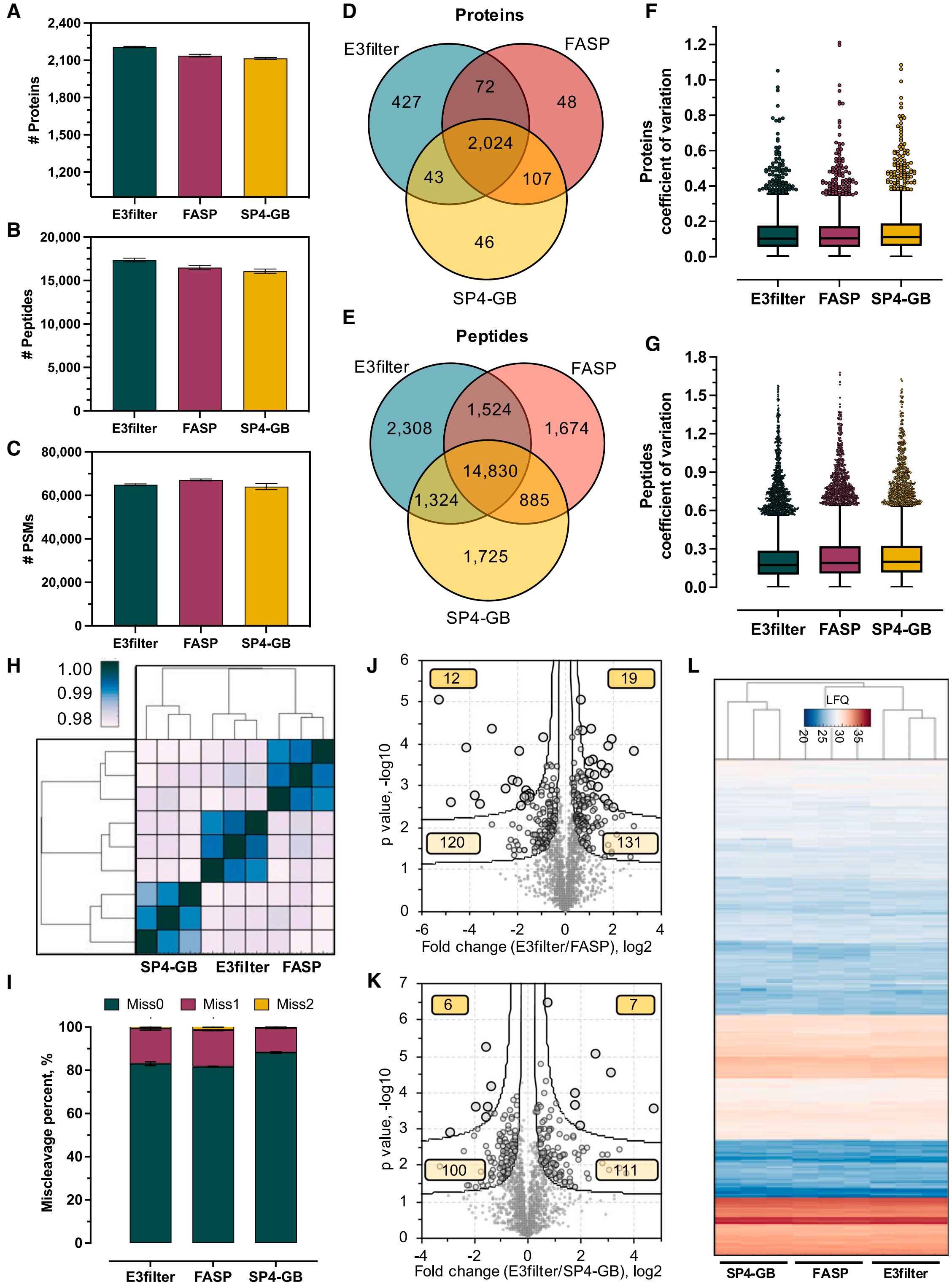

Figure 1. Qualitative and quantitative assessment of the E3filter for *E. coli* proteome analysis

(A–C) Comparison of the number of proteins, peptides, and PSMs between the E3filter, FASP, and SP4-GB approaches. Error bars represent three replicates.
(D and E) Overlapping analyses of proteins and peptides derived from the three methods.

(legend continued on next page)

experiments was on average 0.9910 (±0.0011, n = 3), similar to FASP (0.9907 ± 0.0008) and slightly better than SP4-GB (0.9886 ± 0.0019). The E3filter also quantified more proteins than the other two methods. Over 95% of the total proteins were quantified by at least two replicates, and nearly 90% were quantified by all three of them, which resulted in the largest number of proteins without missing values among the three methods tested here (Figure S1D). The median coefficient of variation (CV) for the E3filter was 10.2%, smaller than FASP (10.5%) and SP4-GB (11.7%), suggesting a generally small variation by E3technology (Figure 1F). Quantitation on the peptide level by the E3filter showed the lowest variations as well (17.3% vs. 19% by FASP and 19.9% by SP4-GB) (Figure 1G). Overall, the profile of the quantified *E. coli* proteome by the E3filter was clustered more closely with FASP than SP4-GB (Figures 1H and 1L), suggesting similarities of the two membrane-based methods. Although pairwise t test revealed some differential proteins between the methods (Figures 1J and 1K), Gene Ontology analyses did not suggest any significant categories in the context of biological process and cellular compartment (data not shown).

E3technology is multi-faceted and widely applicable

We next asked if E3technology could be applied to more complex samples other than the *E. coli* proteome. Here, we examined HEK293 mammalian cells, mouse kidney tissue, and human saliva of various quantities and volumes. In addition to E3filters, we also tested other formats of E3technology by packing the GB membrane into pipette tips (200 μL volume, E3tip), cartridges (1–3 mL volume, E3cartridge), and a 96-well plate (500 μL volume, E3plate).

Mammalian cells

In the context of HEK293 cells, we examined the proteomic performance of E3tips and benchmarked it against another filter-aided method (filter aided sample preparation by easy extraction and digestion, FA-SPEED) that was reported recently.[25] In this method, the cells were lysed with pure TFA and then neutralized and precipitated with acetone, followed by on-membrane cleanup and digestion. We applied the same protocol to the GB membrane. The experimental data indicates that from a 20 μg protein input, E3tips could identify over 5,200 and 39,000 non-redundant protein groups and peptides, respectively, as well as nearly 86,000 peptide features (Figures 2A–2C). These numbers were very reproducible among replicates, and the quantitative reproducibility of the method was excellent as well (average Pearson correlation: 0.99) (Figures 2F and 2G). In terms of digestion efficiency, the percentage of missed cleavages for E3tips was about 10%–13%. When compared to FA-SPEED, the identification rates were nearly the same, with significant overlaps for proteins (93%) and peptides (85%) (Figures 2D and 2E). Meanwhile, the quantitative correlations between the two methods were consistently high, averaging 0.97 for proteins and 0.93 for peptides, respectively (Figure S2A). These data suggest high similarities between the two membrane types. In terms of quantitative variations, the median values of the CV on the protein level were 8.1% for E3tips and 8.5% for FA-SPEED and on the peptide level were 14.0% and 14.3% for the two methods, respectively, suggesting a slight advantage of E3tip over FA-SPEED (Figures 2H and 2I). The minimal number of statistically different proteins, 15 out of over 5,000 hits, as shown in Figure 2J, did not suggest meaningful biological significance after Gene Ontology analysis. Notably, although the FA-SPEED approach generated good-quality data in our study, low recovery and reproducibility have been previously reported.[14]

Kidney tissues

We then evaluated the performance of E3technology using tissue samples, which tend to have a wide dynamic range and high complexity.[26,27] Here, we chose mouse kidney tissues that were derived from one of our previous studies[28] and compared E3tips with the STrap tip (S-tip) method.[8] The latter is based on organic solvent (90% methanol) precipitation of proteins lysed in SDS buffer followed by protein cleanup and digestion on glass fiber filters. We applied the same processing approach to E3tips and S-tips. Our data indicate that the E3tip exceeds the S-tip in terms of protein and peptide identification with triplicate experiments (Figure 3A). Moreover, nearly 90% and 80% of the hits were mutually identified by the two methods (Figure 3C). Quantitative correlations within the E3tip or between the two methods were high as well (Figure 3D). These data indicated the high reproducibility of the E3tip as well as large similarities to the S-Tip method. Although further quantitative analysis suggested some method-specific proteins (permutation false discovery rate [FDR]: 0.05; Figure 3B), these proteins showed only minor variations in most of the cellular compartments, such as the cytoplasm, mitochondrion, and endoplasmic reticulum (Figure 3E).

Human saliva

Large-scale studies, in particular clinical proteomics and biomarker discovery science, require high-throughput sample processing. Thus, we investigated the possibility of multiplexing the E3technology. In this proof-of-concept study, we cast the GB membrane into a deep-well 96-well filter plate and processed human saliva specimens to demonstrate its potential for clinical proteomics analysis (Figures 3F–3J). On average, 1,400 proteins could be identified from each of the five wells (Figure 3F), almost doubling the number of proteins reported by our previous study.[29] Over 85% of the salivary proteins were detected in at least three out of five wells, suggesting minimal variations between the wells (Figure 3G). The successful implementation of the E3plate provided clear evidence for its automation, which is not achievable by centrifugation-based methods,

(F and G) Coefficient of variation of quantified proteins and peptides by three methods.
(H) Heatmap of Pearson correlation between replicate experiments and different methods.
(I) Percentages of missed cleavages. Error bars indicate three replicate experiments.
(J and K) Volcano plot showing significantly differential proteins between E3filter vs. FASP and E3filter vs. SP4-GB. The two curves show FDR = 0.05 and 0.01, respectively. The boxed numbers are significant proteins for each category.
(L) Overall heatmap of all the replicates of the three methods.

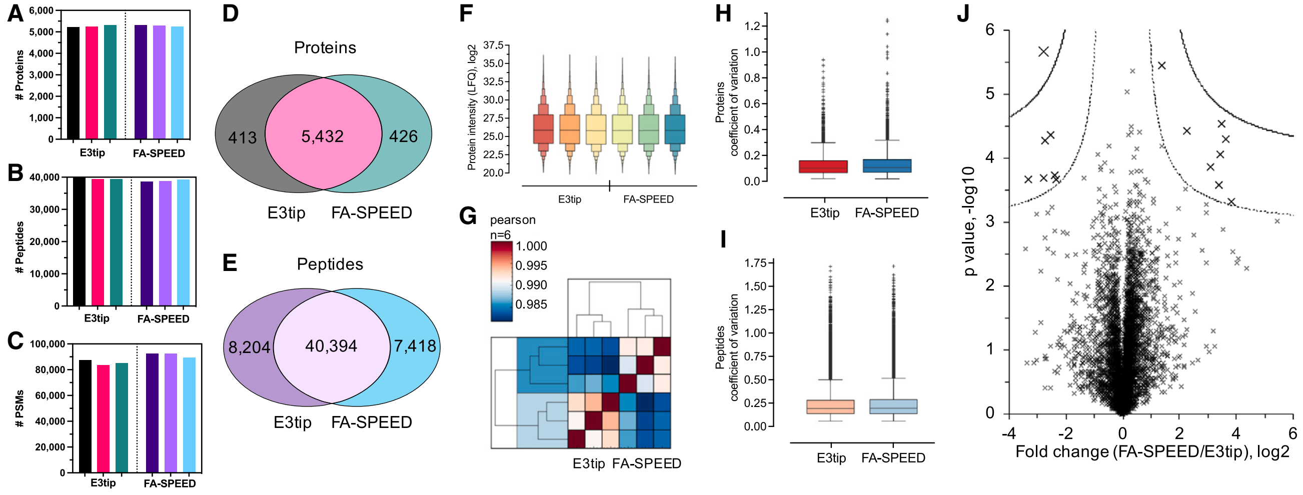

Figure 2. Qualitative and quantitative comparison of the E3tip and FA-SPEED for HEK293 cell proteome analysis

(A–C) Triplicate experiments of identifications of proteins, peptides, and PSMs.

(D and E) Venn diagrams of protein and peptide identifications of the two methods.

(F) Overall protein intensity distribution of the two methods.

(G) Heatmap of Pearson correlations.

(H and I) Protein and peptide coefficient of variation. Data were from triplicate experiments.

(J) Volcano plot shows quantitative comparison of the two methods.

such as SP4. We next asked if E3technology could be used to process diluted samples (e.g., secretome analysis) or samples with large volumes. Here, as a proof of principle, we processed 1 mL of a saliva specimen using a cartridge format of the E3technology and demonstrated an equivalent performance for saliva proteome identification and quantitation (Figures 3F, 3H, and 3I). Functional annotation of the identified saliva proteins was performed by mapping them to two existing databases, OMIM (https://www.omim.org) and the HSP Wiki (https://www.salivaryproteome.org/). The former contains causative genes of a variety of human genetic diseases, and the latter is a collection of human saliva proteins identified by proteomic technologies. Interestingly, almost 93% of the identified saliva proteins from this study were included in the OMIM database, suggesting that E3technology is capable of detecting marker proteins for clinical diagnosis (Figure 3J). In addition, nearly 40% of them were still new to the HSP Wiki database, implying reasonably good coverage of E3technology for saliva proteome analysis.

Enhanced E3technology (E4technology) enables low-input and low-cell proteomics

Despite recent progress in single-vessel approaches that aim to reduce sample loss and increase proteomic sensitivity,[30] analyzing quantity-limited samples such as biopsy, rare cells, or precious biospecimens is still challenging. One of the possible reasons is that these methods suffer from poor recovery, have a limited choice of lysing reagents, or require additional sample processing outside of the device, thus compromising the sensitivity. Here, we examined if E3technology could be used to process submicrogram protein samples. We chose the E3tip as a representative method due to its relatively small contact surface and process volume. Our data showed that, from 1 μg input of a whole HEK293 cell lysate, the E3tip out-performed the S-tip (Figure 4A), enabling the identification of over 3,700 unique proteins and 20,000 peptides, similar to the identification output of SP3 and in-StageTip (iST) methods using the same amount of lysate input.[30]

We then took a step further and asked if E3technology could be used as a single-vessel approach to perform complete sample preparation in one device. Here, we adopted the in-cell digestion ideas reported recently[31,32] and also took advantage of the Empore technique to create a multi-functional GB|C18 membrane. We then investigated if the C18-enhanced E3technology (named E4technology) could facilitate low-cell analysis. In this experiment, the cells were fixed by methanol onto the E4filters, which were then subjected to on-filter in-cell (OFIC) digestion directly. Afterward, the resulting peptides were conveniently desalted through the E4filters with C18 functionality, leading to LCMS-ready samples after lyophilization. Since no cell lysis was involved, and all the processing steps were carried out in the same device, such an OFIC approach should minimize sample loss to the largest extent. In our initial experiment that started with roughly 25,000 Jurkat cells, we were able to identify, on average, over 4,300 proteins and nearly 30,000 peptides. By contrast, when the same number of cells was first lysed with SDS buffer and then digested using the E3 procedures established above, 20% fewer protein and 50% fewer peptide hits were identified (Figure 4C). These data suggested that conventional "cell lysis-protein digestion" methods are associated with significant sample losses, which would be detrimental to low-load proteomics analysis.

In further experiments, our data showed that the identification rate remained consistent when the starting number of Jurkat cells was reduced by half (to 10,000 cells), whereas it dropped

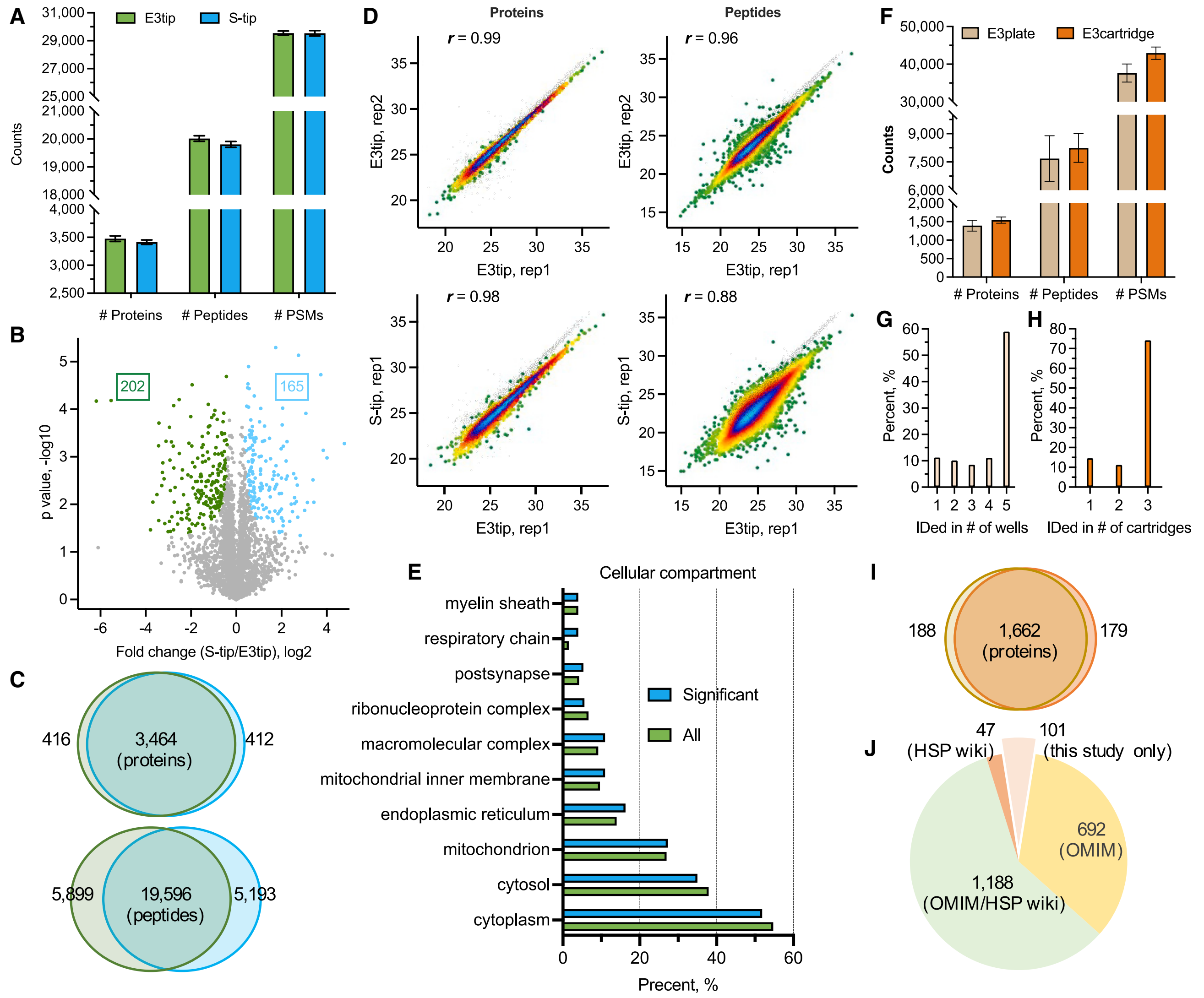

Figure 3. Applying E3technology to kidney and saliva specimens

(A) Histogram shows comparison of the total number of identified kidney proteins and peptides. Data were from triplicate experiments of each method.
(B) Volcano plot shows quantitative comparison of the two methods. Colored dots represent significant proteins (permutation FDR = 0.05).
(C) Overlaps of protein and peptide identifications between the two methods.
(D) Correlation analyses of the two methods. Protein- and peptide-level density plots of replicate experiments within the E3tip (top two) and between the E3tip and S-Tip methods (bottom two), respectively. Two representative E3tip experiments (rep1 and rep2) and one S-tip experiment are plotted. Pearson correlation values are shown on the top left corner of the plots.
(E) Gene Ontology cellular compartment analysis of significant proteins (from plot B) and the overall quantified kidney proteins.
(F) Illustration of the number of saliva protein, peptide, and PSMs identifications by E3plate and E3cartridge. Five replicate wells and three replicate cartridges were used for this experiment.
(G and H) Frequency of protein identifications.
(I) Protein overlapping between E3plate and E3cartridge.
(J) Classifying saliva protein identified from study.

to 1,300 proteins and 3,500 peptides when the starting material was 2,500 cells (Figure 4B). Additionally, we examined the OFIC digestion method using yeast cells, which usually require harsh lysis conditions for protein extraction. In our experiments, interestingly, the yeast cells became completely permeable and digestible by trypsin after a simple fixing step by methanol. From around 1.6 million yeast cells (the amount of protein contents equivalent to 25,000 HeLa cells), we were able to identify nearly 3,500 proteins and over 32,000 peptides. When the cell number reduced by half, the identification rates of proteins and peptides were 2,472 and 18,847 (90 min LCMS run), respectively. When the cells reduced down to 160,000 (equivalent to 2,500 HeLa cells), the numbers of proteins and peptides were 1,160 and 6,097, respectively (Figure 4D). These data suggest

CellPress
OPEN ACCESS

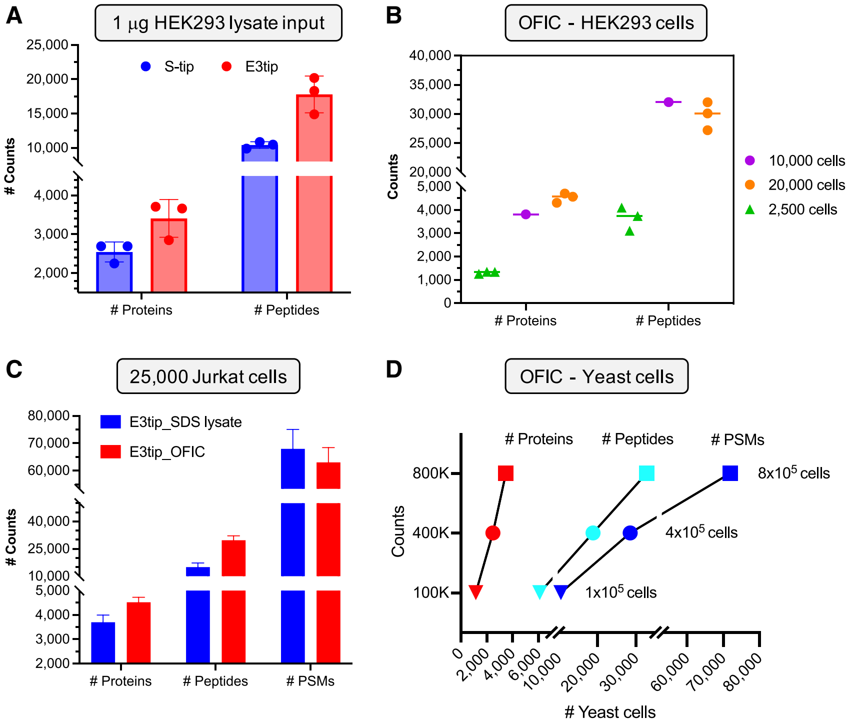

Figure 4. Applying E3technology to low-input samples

(A) Comparison of protein and peptide identifications from 1 µg of HEK293 cell lysate. Error bars indicate triplicate experiments.

(B and D) Assessment of OFIC digestion using (B) mammalian cells and (D) yeast cells.

(C) Comparison of on-filter digestion using SDS lysate or intact cells. Error bars represent triplicate experiments.

that OFIC-based E4technology is a highly efficient way to profile the intact cell proteome with minimal input.

Applying E3technology to study RBPs

Circular RNAs (circRNAs) are a recently appreciated class of regulatory RNAs, which regulate gene expression primarily by either sponging microRNAs (miRNAs) or interacting with RBPs.[33] The interactions of circRNAs with RBPs are less well studied as compared to their binding to miRNAs, mainly due to the lack of robust and reliable technologies to identify their interactions. Affinity purification (AP) followed by MS-based quantitative proteomic approaches has been utilized widely as an unbiased way to identify protein-RNA interactions.[34–36] However, most of the existing approaches rely on gel-based or in-solution sample processing, which is either time consuming or requires a large amount of starting material for the pull-down assays. Owing to the low-volume processing and loss-less characteristics of the E3tip, we anticipated that it could be used to analyze precious or low-input samples with high sensitivity. In this study, we applied the E3tip to explore RBPs binding with circRNA. We utilized circNFIX (hsa_circ_0005660) as a model system, which was reported recently to inhibit cardiomyocyte proliferation and recovery through its interaction with Y-box binding protein 1 (YBX1).[37] We also included the linear isoform of nuclear factor I X (NFIX) in this experiment as a control, since its interaction with YBX1 is not known and the functional variations of the circular and linear NFIX in cancer cells are not yet well studied. We adopted the CRISPR-assisted detection of RNA-protein interactions (CARPID) method,[38] utilizing guide RNAs that were specific for both linear and circular NFIX in a colorectal adenocarcinoma cell line, DLD-1 cells, as outlined in Figure 5A. Three biological replicates were performed for each pull-down experiment.

Starting with around 1.5 × 10^{6} cells, we were able to quantify over 3,100 proteins from the pull-down complex at 1% FDR on both the protein and peptide levels. From enrichment analyses, we found that YBX1 was among the significantly (fold change > 1.5, $p < 0.05$) differential proteins identified with circNFIX over control pull-down (Figures 5B, S3A, and S3B). YBX1 is a known RBP and has been shown to play critical roles in the post-transcriptional regulation of epidermal homeostasis,[39] brown adipogenesis and thermogenesis,[40] and cardiac regeneration.[37] It is also a known prognostic marker of a variety of cancers.[41] In our experiment using colorectal cancer cells, we performed simultaneous RNA imaging and immunofluorescence to validate the interaction between circNFIX and YBX1. We utilized the circular fluorescence *in situ* hybridization method that enables simultaneous imaging of linear and circular isoforms of an RNA.[42] In this method, two sets of probes (PL [probe linear] and PC [probe circular]; Figures S3C–S3H) are used. The PL probe set targets exon 11 of NFIX that is specific to the linear isoform of NFIX, whereas probe set PC binds to exon 2, which is common to both linear and circular isoforms. Linear NFIX would be located where the signals of both PL and PC probes colocalize since linear NFIX contains both exons, whereas circNFIX would be located only if there is a signal from the PC probes. Using a custom written MATLAB program to determine colocalization between probe signals, we observed that linear and circNFIX were colocalizing with YBX-1 (Figures 5C and S3). We calculated the proportion of colocalized RNA from the total RNA of that subtype and found that circNFIX had significantly higher colocalization with YBX-1 as compared to linear NFIX (Figure 5D). These data are consistent with the findings from the MS-based approach and suggest the usefulness of the E3tip to analyze low-input samples such as protein complexes. We further examined other potential RBPs from this experiment and found that among the proteins that were highly enriched in circRNA pull-down compared to the control and linear RNA complexes, a large number of them were either RBPs or predicted to be associated with transcription regulation. For instance, transcriptional coactivator YAP1 protein (YAP1) is a known transcription regulator that is heavily involved in the Hippo

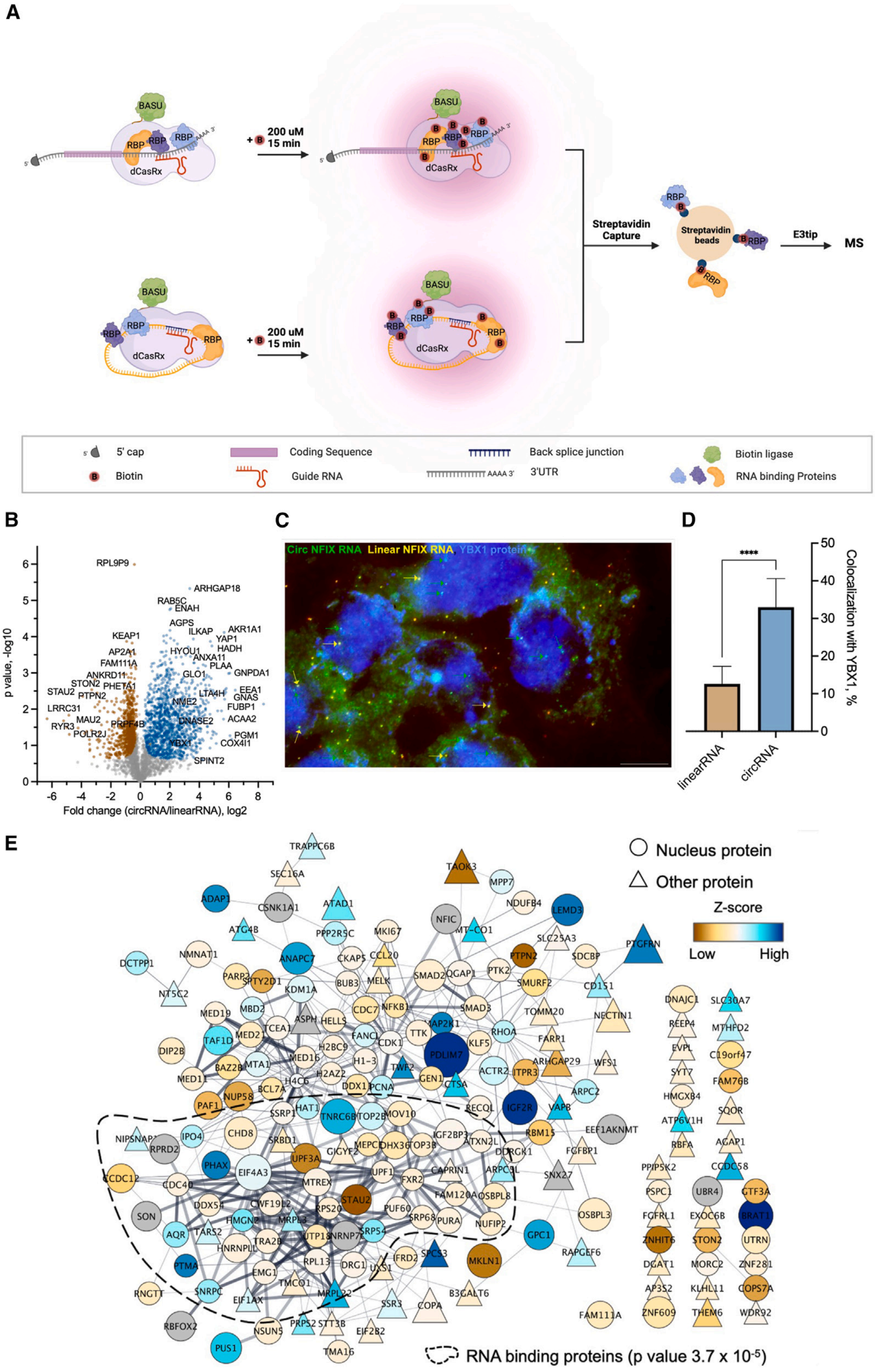

(legend on next page)

signaling pathway, particularly during cancer development.[43] ATP-dependent RNA helicase DDX42 protein is predicted to be an RBP and has shown experimental evidence in colorectal cancer progression.[44] Ro 60-kDa autoantigen is a known RBP that functions in non-coding RNA quality control while trafficking between the nucleus and cytoplasm.[45] To simply visualize the proteins that are significantly associated with NFIX, we took the top 200 significant (up- and down-regulated) proteins quantified from the circRNA-linear RNA comparison to build a network based on *in silico* analysis or experimental evidence in the STRING database (Figure 5E). This protein map showed that the majority (>85%) of them are nuclear proteins, including a network of RBPs, which certainly makes biological sense and is well in line with our experimental design. In summary, the data from this proof-of-principle study further highlight the specificity and high sensitivity of our E3technology for AP-MS analysis.

Applying E4technology to profile intact bacterial cell proteome

Iron-oxidizing bacteria are found in wide-spread environments, including agricultural, acid mine drainage, and water treatment systems, where they influence many biogeochemical cycles.[46–48] Despite their prevalence, knowledge of microbial iron oxidation mechanisms remains limited due to difficulties culturing and analyzing these organisms. *Sideroxydans lithotrophicus* ES-1 (hereafter ES-1) is a robust, facultative iron oxidizer with a sequenced, closed genome encoding extensive metabolic versatility through multiple, putative enzymatic pathways for autotrophic growth on thiosulfate or Fe(II).[49–51] ES-1 has been a known model organism to study the physiology of a facultative iron oxidizer and identify pathways responsible for growth on different substrates through transcriptomics and proteomics. However, there are still technical challenges to overcome during sample processing. For instance, ES-1 cells typically exhibit low density in liquid cultures (10^7–10^8 cells/mL),[51] which require large volumes of cultures in order to obtain sufficient biomass for protein extraction. ES-1 cells also do not easily pellet during normal centrifugation. Thus, previous attempts to isolate DNA/RNA or proteins from ES-1 involved the concentration of cells on membranes (e.g., polyethersulfone [PES], nitrocellulose), which were then cut into small pieces, followed by one-pot on-membrane cell lysis.[51,52] Although the procedure worked well for RNA isolation and transcriptome analysis, it was not applicable to proteomics, as it created severe polymer contamination to MS-based detection (Figure S4A).

Here, we employed the E4cartridge to characterize the ES-1 cell proteome during growth with thiosulfate. Using 16–24 mL of cell culture ($\sim$1–1.5 $\times$ 10^9 cells), less than 1/10 of the previous cell input,[52] we were able to identify around 13,000 unique peptide hits and over 2,200 non-redundant protein groups, which nearly doubled the number of identifications from the previous study that was based on the SDS lysis method.[52] Such a high identification rate from the low amount of starting material, not only highlights the effectiveness of the OFIC digestion approach but also emphasizes the loss-less and contamination-free characteristics of the E4technology. To better evaluate the proteome data obtained above, we took the transcriptomics information of the same cells and performed direct comparisons. While the RNA sequencing approach unsurprisingly detected nearly the entire genome, MS-based proteomics identified over 76% of protein-coding genes (Figure 6A), suggesting a good depth of sampling. The dynamic range of the detected transcripts spanned about four orders of magnitude, whereas that of proteins spanned at least one magnitude higher, which explains in part the challenges of identifying more proteins, especially those in low abundance. To better visualize the differences between mRNA and protein expression in the ES-1 cells, we plotted the transcripts per million-based RNA intensities vs. the intensity-based absolute quantification-based protein intensities. The mRNA-protein correlation was generally good, with a Spearman's correlation of 0.56 (Figure 6C), similar to that of mammalian cells and tissues derived from ultra-deep transcriptome and proteome studies.[27,53] On the other hand, the plot of the ranked orders of proteins and transcripts indicated much wider variations. While 21 and 159 of the most abundant mRNAs were 25% and 50% of the quantity of all transcripts, respectively, only 3 proteins represented a quarter of the total protein intensities already (Figure 6D). Slit_2477 (porin), Slit_0538 (pilin), and Slit_0967 (cold-shock DNA-binding domain protein) were among the top 10 most abundant proteins and were also seen in the top 10 list of transcripts. These proteins are likely associated with some of the fundamental regulations in ES-1 cells, such as DNA binding, cell motility and anchoring, and/or fatty acid biosynthesis.[50,54,55] However, experimental evidence of function is lacking for most of the proteins of ES-1. Therefore, the exact functions of these highly expressed proteins warrant further investigation. Interestingly, both porin and pilin are transmembrane proteins, and our proteomic experiment identified multiple peptides from the beta-barrel and alpha-helix regions. Meanwhile, around 25.4% of the identified proteins were predicted to contain one or more transmembrane helices,

Figure 5. Applying the E3tip to AP-MS analysis

(A) Illustrative workflow of CARPID-base assay. The complex of dCasRx-BASU (deactivated Cas9 fused with biotin ligase) is targeted by a guide RNA designed to bind the 3′ UTR of linear RNA or the back splice junction of circRNA targets, respectively. When cells are incubated with biotin, the biotin ligase biotinylates RNA-binding proteins that are associated with the RNA target. These biotinylated proteins are then captured using streptavidin beads and processed by E3tip-LCMS.

(B) Quantitative comparison of the circRNA pull-down with linear RNA pull-down complexes. The curve indicates the permutation FDR (0.05).

(C) A representative image of DLD-1 cells hybridized with a mixture of two probes (a probe set exclusive for a linear RNA exon labeled with Texas red and a probe set for an exon that yields circRNA labeled with Cy5) followed by immunofluorescence for YBX-1 protein using Alexa 488-conjugated antibody. The three channels were merged to show linear and circular NFIX colocalization with YBX1. Full-length linear RNA is pseudo-colored as yellow spots (had signal for both probe sets), while the circRNA signal is pseudo-colored green, and the YBX1 protein is represented in blue. Green arrows point to circular and yellow arrows point to linear RNA colocalizing with YBX1 protein.

(D) Quantification of RNA colocalization with YBX-1 protein represented by average percentage of linear RNA and circRNA out of total of each subtype per cell. At least 40 cells were counted for each; error bars represent 95% confidence interval, and ***p < 0.001.

(E) Protein networks derived from Cytoscape using the top 200 significant proteins from the plot in (B).

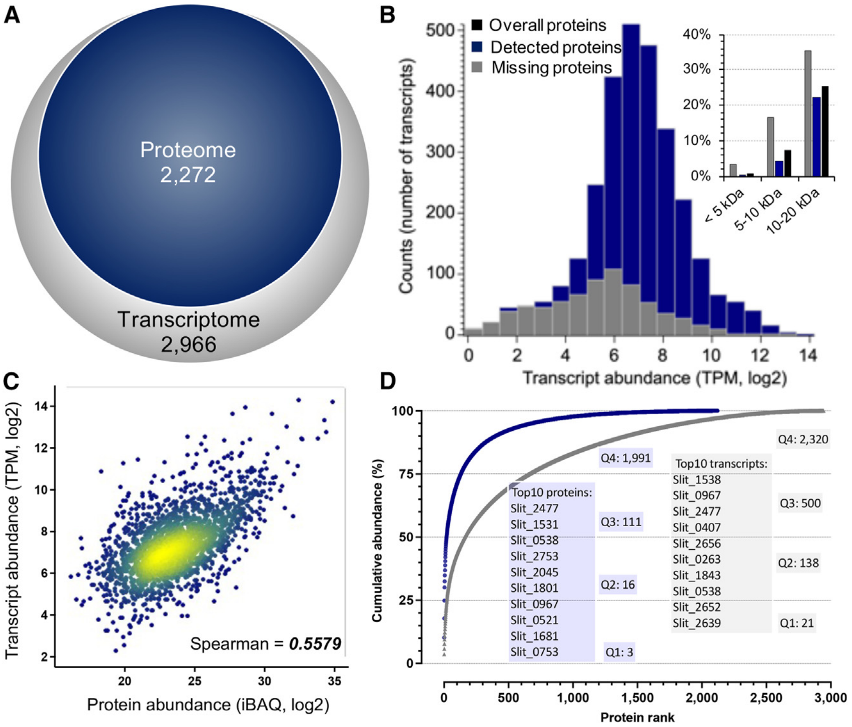

Figure 6. E4technology enables intact bacterial cell proteome analysis

(A) Transcriptomic and proteomic coverage of the *Sideroxydans lithotrophicus* ES-1 cells.

(B) Abundance distribution of all the ES-1 transcripts. Color-coded bars highlight the corresponding proteins detected (blue) or missed (gray) by proteomics. The inset displays the molecular weight distribution of the protein in each category, including the overall predicted proteome (black). An extended figure is included in Figure S7.

(C) Intensity correlation of transcripts and their corresponding proteins.

(D) Dynamic range analyses of the transcriptome (gray) and proteome (blue) of the ES-1 cells. The number of proteins in each quarter and the top 10 most abundant transcripts and proteomes are listed.

consistent with the trend of the entire proteome (26.3%). These data are consistent with previous findings[31] and again highlight that the in-cell digestion approach is unbiased toward membrane protein identifications.

Regarding the "missing proteins," the inability to identify them by the E4cartridge may be simply because their expressions are below the detection limit of MS. Indeed, the majority of the low-abundance transcripts did not translate to detectable proteins by MS (Figure 6B). However, some of the proteins were still missing on mass spectrometer despite the high expression of their mRNAs. Their absence may be associated with the difficulties of digestion or peptide extraction from those proteins, leading to few MS-compatible peptides. For instance, nearly 20% of the missing proteins are less than 10 kDa (Figure 6B, inset), including 26 (out of 27 from the total proteome) that are <5 kDa. An uncharacterized gene, Slit_2652, ranked in the top 10 with high mRNA intensity, whereas its protein form was undetected, likely due to the extremely small size (3.8 kDa) and absence of tryptic cleavage sites. In summary, this application highlights the simplicity and robustness of the E4technology for intact cell proteome analysis. For the first time, the application also provides a high-coverage proteome reference map of *S. lithotrophicus* ES-1 as well as a systematic examination of the correlation between protein and mRNA expression, which will assist mechanistic studies of iron oxidation in the future.

DISCUSSION

Current proteomics analysis has been largely hindered by the lack of a universal sample preparation methodology that combines robustness, cost effectiveness, and high efficiency for cell lysis, protein cleanup, and digestion. In this study, we developed a GB membrane and new devices and systematically investigated their feasibility for proteomics sample preparation. The data from our study demonstrate that the GB membrane can serve as an efficient, effective, and economical medium for sample processing prior to MS analysis. Particularly, E3filters out-performed FASP and SP4-GB methods in the context of proteome-wide identification and quantitation of *E.coli* cells. FASP has been one of the most widely adopted preparation methods in the past decade by the proteomics community,[6] whereas the SP4-GB method was reported only recently.[14] Its principle is similar to the SP3 approach,[9] but instead of using magnetic beads, it uses inert GBs as support for protein precipitation. Interestingly, the report claimed only marginal advantages of the GBs over a bead-free method and stated that GBs could even be omitted altogether. We argue that the approach of using GBs for proteomics sample preparation was underestimated and its potential could be further explored when they are immobilized. The advantages of using fixed beads are obvious, as it eliminates the risks of sample loss from non-elegant pipetting,[14] incomplete resuspension of dense bead aggregates,[16] or beads that stick to pipette tips or tube walls.[17] Notably, in our SP4 experiments, we consistently detected a significant number of unprecipitated proteins from the supernatant and/or the wash solutions (data not shown), which again raised concerns of contamination and/or sample loss.

In the FA-SPEED method, cells are lysed with pure TFA, introducing no detergents to the cell lysate.[25] However, it is not necessarily universal, as the authors suggested. In our experiments, TFA did not provide good protein yields when processing real fecal specimens and some filamentous bacteria such as *Leptothrix cholodnii*, whereas SDS-based buffer did (data not shown). The TFA-derived lysate is incompatible with *in vitro* AP or protein-protein interaction studies either. In addition, the protocol involves a series of neutralization and dilution steps; thus, the large final volume raises concerns of sample

loss from low input or small number of cells. In this study, we compared TFA lysis experiments with and without neutralization. Our data showed no qualitative or quantitative differentiation between the two procedures (Figures S2B and S2C), suggesting that adjustments or further optimization may be necessary in order to better fit different applications. Regarding the glass fiber-based S-tip and its commercialized form, the S-Trap filter, a distinct advantage of the methods is the fast liquid transfer, which makes it a rapid approach for general proteomics applications.[56] However, unlike Empore membranes, the rigidity of the glass fiber membrane is not optimal for cutting small discs and packing into pipette tips. Meanwhile, the cost of commercial S-Trap devices is comparably high. Therefore, the E3filter presents an equivalent yet economical alternative to the STrap approach. We further showcased the practical applicability of the E3technology to biospecimen analysis and clinical proteomics. Such studies tend to include large cohorts and sometimes difficult-to-obtain samples.[57] The E3plate approach offers a cost-effective and reliable alternative to sample preparation for clinical proteomics. Overall, in our facility, we have utilized E3 devices to process a few hundred saliva, plasma, tissue, and fecal specimens so far and consistently obtained good-quality data.

Low-cell and single-cell proteomics have gained great attention recently. One of the keys to success is to minimize sample loss and maintain maximal sensitivity.[58] Currently available approaches typically require specialized equipment that suffer from labor intensiveness and/or high cost,[59–61] which make them challenging for general biological laboratories to perform.[62] Notably, recent advances have been seen by incorporating novel trap columns,[63] simplifying sample processing,[64] in-line desalting,[65] or utilizing low-cost cell dispensers,[66] all of which greatly facilitated the low-/single-cell analysis. The OFIC digestion-based E4technology further simplifies the processing and provides pipette tip-based, easily accessible devices to general research laboratories. Intact cells could be processed directly in this single-filter device without any tube-to-tube transfer or pipetting, thus minimizing the unspecific adsorption to plastic surfaces. Nucleic acid contamination has been reported as a major concern during the tube-based in-cell digestion process,[31] whereas the on-filter processing strategy eliminates the above concerns naturally. Regarding the concern of potential trypsin retention to the C18 materials, our data did not suggest any side effects of the on-membrane digestion methods in the context of protein and peptide identifications and digestion efficiencies (Figures S4B–S4D).

We further asked if the E3 and E4 technologies could be used to answer real biological questions. We employed the E3tip to process protein complex samples derived from streptavidin-based AP. We achieved a high identification rate and identified known interactors as well as a large number of potentially new partners to circNFIX and linear NFIX, which certainly warrant future functional investigations. Meanwhile, we utilized E4technology to profile the proteome of intact bacterial cells. The OFIC processing led to LCMS-ready samples directly. The merits of E4technology could be further amplified when adding fractionation or enrichment to the process, which is not achievable by any existing low-cell or single-cell proteomic methods.

The proteomics community has advanced drastically in the past few years due to the development of new MS acquisition methods, MS instrumentation, and novel concept LC systems.[67,68] Nevertheless, high-quality protein digestion is still a prerequisite for any successful bottom-up proteomics analysis. The technologies developed from our study have been demonstrated to be efficient, effective, and economical alternatives to existing methods in the field. As the E3 and E4 filter devices are becoming commercially available, we anticipate that these easily accessible and much affordable technologies will be widely adopted by the proteomics community.

Limitations of the study

The E3 and E4 filters originally used in this study were manually assembled, which might be inconvenient to some proteomics laboratories. During the revisions of the manuscript, the filters have become commercially available. Please refer to the key resources table for the ready-to-go products. In addition, the identification rates of the E4tips for low-cell samples (Figures 4B–4D) appeared to be low in comparison to some other dedicated single-cell proteomics studies, where cutting-edge cell handling techniques and optimal LCMS configurations were used for single-cell analysis.[65,69,70,71] The fact is that although E4technology bypasses the cell lysis step and performs peptide cleanup/desalting in the same device, the following "elution-drying-resuspension" procedures before LCMS injection increase the risk of sample loss. We anticipate that improved proteome coverage could be achieved when applying the E4tip to other LC systems such as Evosep One, which could elute desalted peptides off the tip directly for LCMS acquisition.[65]

STAR★METHODS

Detailed methods are provided in the online version of this paper and include the following:

- KEY RESOURCES TABLE
- RESOURCE AVAILABILITY
  - Lead contact
  - Materials availability
  - Data and code availability
- EXPERIMENTAL MODEL AND STUDY PARTICIPANT DETAILS
- METHOD DETAILS
  - Cell culture, and tissue and specimen collection
  - E3filter experiments and SP4-GB, FASP digestion of *E. coli* proteins
  - E3tip experiments and FA-SPEED digestion of HEK293 proteins
  - E3tip experiments and S-Tip kidney protein digestion
  - E3cartridge and E3plate saliva experiments
  - E4tip and on-filter in-cell (OFIC) digestion experiments
  - *Sideroxydans lithotrophicus* ES-1 cell culture, collection, and OFIC sample preparation
  - LC-MS/MS analysis
  - Proteome identification and quantitation
  - Experiment of studying RNA-binding proteins
  - CARPID
  - CircFISH and immunofluorescence
  - Fluorescence imaging and analysis
- QUANTIFICATION AND STATISTICAL ANALYSIS

SUPPLEMENTAL INFORMATION

Supplemental information can be found online at https://doi.org/10.1016/j.crmeth.2024.100796.

ACKNOWLEDGMENTS

We gratefully acknowledge support from the National Institute of General Medical Sciences (NIGMS) under award number P20GM104316 for the MS instrument, the National Science Foundation (NSF) under award number 2242127 (M.B.), NSF BIO-1817651 (C.S.C.), and NIH R01GM129468 (Z.Z.). We would like to thank Zaining Yun in the Chemical & Biomolecular Engineering Department for providing the Jurkat cells. We also thank Dr. Yong Zhao in the W.M. Keck Center for the microscopy analysis. The graphical abstract was created with BioRender.

AUTHOR CONTRIBUTIONS

K.R.M., H.T.L., and C.Y. performed some of the preparation for the method evaluation experiments; A.A. and M.B. designed the RNA probes, performed CARPID and AP experiments, and interpreted data; J.L.K. and C.S.C. performed the ES-1 cell culture experiments and interpreted the transcriptomic data; G.L. and X.Z. assisted with manufacturing of GB membranes and guided the design of the E3 and E4 filters; Z.Z. and P.N.A.-O. assisted with the cell experiments and oversaw data collection and analysis; and Y.Y. conceived the study, collected data for patent application, performed the majority of sample preparations, analyzed data, and wrote the manuscript with input from all other authors. All authors provided feedback and edited the manuscript.

DECLARATION OF INTERESTS

G.L. and X.Z. are employees of CDS Analytical LLC. Y.Y. is a paid consultant of CDS Analytical LLC. Y.Y. is a named inventor on a patent application (PCT/US2023/020215) for the technologies developed in this study. The patent was published under the publication number WO2023/212205 and has been licensed exclusively to CDS Analytical LLC through the University of Delaware.

Received: January 19, 2024
Revised: March 21, 2024
Accepted: May 20, 2024
Published: June 11, 2024

REFERENCES

1. Zhang, Y., Fonslow, B.R., Shan, B., Baek, M.-C., and Yates, J.R. (2013). Protein Analysis by Shotgun/Bottom-up Proteomics. Chem. Rev. *113*, 2343–2394. https://doi.org/10.1021/cr3003533.
2. Varnavides, G., Madern, M., Anrather, D., Hartl, N., Reiter, W., and Hartl, M. (2022). In Search of a Universal Method: A Comparative Survey of Bottom-Up Proteomics Sample Preparation Methods. J. Proteome Res. *21*, 2397–2411. https://doi.org/10.1021/acs.jproteome.2c00265.
3. Feist, P., and Hummon, A.B. (2015). Proteomic Challenges: Sample Preparation Techniques for Microgram-Quantity Protein Analysis from Biological Samples. Int. J. Mol. Sci. *16*, 3537–3563. https://doi.org/10.3390/ijms16023537.
4. Shevchenko, A., Wilm, M., Vorm, O., and Mann, M. (1996). Mass spectrometric sequencing of proteins silver-stained polyacrylamide gels. Anal. Chem. *68*, 850–858. https://doi.org/10.1021/ac950914h.
5. Manza, L.L., Stamer, S.L., Ham, A.-J.L., Codreanu, S.G., and Liebler, D.C. (2005). Sample preparation and digestion for proteomic analyses using spin filters. Proteomics *5*, 1742–1745. https://doi.org/10.1002/pmic.200401063.
6. Wiśniewski, J.R., Zougman, A., Nagaraj, N., and Mann, M. (2009). Universal sample preparation method for proteome analysis. Nat. Methods *6*, 359–362. https://doi.org/10.1038/nmeth.1322.
7. Berger, S.T., Ahmed, S., Muntel, J., Cuevas Polo, N., Bachur, R., Kentsis, A., Steen, J., and Steen, H. (2015). MStern Blotting–High Throughput Polyvinylidene Fluoride (PVDF) Membrane-Based Proteomic Sample Preparation for 96-Well Plates*[S]. Mol. Cell. Proteomics *14*, 2814–2823. https://doi.org/10.1074/mcp.O115.049650.
8. Zougman, A., Selby, P.J., and Banks, R.E. (2014). Suspension trapping (STrap) sample preparation method for bottom-up proteomics analysis. Proteomics *14*, 1006. https://doi.org/10.1002/pmic.201300553.
9. Hughes, C.S., Foehr, S., Garfield, D.A., Furlong, E.E., Steinmetz, L.M., and Krijgsveld, J. (2014). Ultrasensitive proteome analysis using paramagnetic bead technology. Mol. Syst. Biol. *10*, 757. https://doi.org/10.15252/msb.20145625.
10. Kulak, N.A., Pichler, G., Paron, I., Nagaraj, N., and Mann, M. (2014). Minimal, encapsulated proteomic-sample processing applied to copy-number estimation in eukaryotic cells. Nat. Methods *11*, 319–324. https://doi.org/10.1038/nmeth.2834.
11. Duong, V.-A., and Lee, H. (2023). Bottom-Up Proteomics: Advancements in Sample Preparation. Int. J. Mol. Sci. *24*, 5350. https://doi.org/10.3390/ijms24065350.
12. Nickerson, J.L., and Doucette, A.A. (2020). Rapid and Quantitative Protein Precipitation for Proteome Analysis by Mass Spectrometry. J. Proteome Res. *19*, 2035–2042. https://doi.org/10.1021/acs.jproteome.9b00867.
13. Jiang, L., He, L., and Fountoulakis, M. (2004). Comparison of protein precipitation methods for sample preparation prior to proteomic analysis. J. Chromatogr. A *1023*, 317–320. https://doi.org/10.1016/j.chroma.2003.10.029.
14. Johnston, H.E., Yadav, K., Kirkpatrick, J.M., Biggs, G.S., Oxley, D., Kramer, H.B., and Samant, R.S. (2022). Solvent Precipitation SP3 (SP4) Enhances Recovery for Proteomics Sample Preparation without Magnetic Beads. Anal. Chem. *94*, 10320–10328. https://doi.org/10.1021/acs.analchem.1c04200.
15. Batth, T.S., Tollenaere, M.X., Rüther, P., Gonzalez-Franquesa, A., Prabhakar, B.S., Bekker-Jensen, S., Deshmukh, A.S., and Olsen, J.V. (2019). Protein Aggregation Capture on Microparticles Enables Multipurpose Proteomics Sample Preparation. Mol. Cell. Proteomics *18*, 1027–1035. https://doi.org/10.1074/mcp.TIR118.001270.
16. Hughes, C.S., Moggridge, S., Müller, T., Sorensen, P.H., Morin, G.B., and Krijgsveld, J. (2019). Single-pot, solid-phase-enhanced sample preparation for proteomics experiments. Nat. Protoc. *14*, 68–85. https://doi.org/10.1038/s41596-018-0082-x.
17. Müller, T., Kalxdorf, M., Longuespée, R., Kazdal, D.N., Stenzinger, A., and Krijgsveld, J. (2020). Automated sample preparation with SP3 for low-input clinical proteomics. Mol. Syst. Biol. *16*, e9111. https://doi.org/10.15252/msb.20199111.
18. Rappsilber, J., Ishihama, Y., and Mann, M. (2003). Stop and Go Extraction Tips for Matrix-Assisted Laser Desorption/Ionization, Nanoelectrospray, and LC/MS Sample Pretreatment in Proteomics. Anal. Chem. *75*, 663–670. https://doi.org/10.1021/ac026117i.
19. Wiśniewski, J.R., Zougman, A., and Mann, M. (2009). Combination of FASP and StageTip-Based Fractionation Allows In-Depth Analysis of the Hippocampal Membrane Proteome. J. Proteome Res. *8*, 5674–5678. https://doi.org/10.1021/pr900748n.
20. Dimayacyac-Esleta, B.R.T., Tsai, C.-F., Kitata, R.B., Lin, P.-Y., Choong, W.-K., Lin, T.-D., Wang, Y.-T., Weng, S.-H., Yang, P.-C., Arco, S.D., et al. (2015). Rapid High-pH Reverse Phase StageTip for Sensitive Small-Scale Membrane Proteomic Profiling. Anal. Chem. *87*, 12016–12023. https://doi.org/10.1021/acs.analchem.5b03639.
21. Chen, W., Wang, S., Adhikari, S., Deng, Z., Wang, L., Chen, L., Ke, M., Yang, P., and Tian, R. (2016). Simple and Integrated Spintip-Based Technology Applied for Deep Proteome Profiling. Anal. Chem. *88*, 4864–4871. https://doi.org/10.1021/acs.analchem.6b00631.
22. Mao, Y., Chen, P., Ke, M., Chen, X., Ji, S., Chen, W., and Tian, R. (2021). Fully Integrated and Multiplexed Sample Preparation Technology for Sensitive Interactome Profiling. Anal. Chem. *93*, 3026–3034. https://doi.org/10.1021/acs.analchem.0c05076.
23. Rappsilber, J., Mann, M., and Ishihama, Y. (2007). Protocol for micro-purification, enrichment, pre-fractionation and storage of peptides for

proteomics using StageTips. Nat. Protoc. *2*, 1896–1906. https://doi.org/10.1038/nprot.2007.261.

24. Lin, Y.H., Eguez, R.V., Vashee, I., and Yu, Y. (2021). Lab-on-a-Filter Techniques for Economical, Effective, and Flexible Proteome Analysis. In Methods in Molecular Biology (Humana Press Inc.), pp. 25–34. https://doi.org/10.1007/978-1-0716-1186-9_3.
25. Doellinger, J., Schneider, A., Hoeller, M., and Lasch, P. (2020). Sample Preparation by Easy Extraction and Digestion (SPEED) - A Universal, Rapid, and Detergent-free Protocol for Proteomics Based on Acid Extraction. Mol. Cell. Proteomics *19*, 209–222. https://doi.org/10.1074/mcp.TIR119.001616.
26. Chandramouli, K., and Qian, P.-Y. (2009). Proteomics: challenges, techniques and possibilities to overcome biological sample complexity. Hum. Genomics Proteomics. *2009*, 239204. https://doi.org/10.4061/2009/239204.
27. Wang, D., Eraslan, B., Wieland, T., Hallström, B., Hopf, T., Zolg, D.P., Zecha, J., Asplund, A., Li, L.-H., Meng, C., et al. (2019). A deep proteome and transcriptome abundance atlas of 29 healthy human tissues. Mol. Syst. Biol. *15*, e8503. https://doi.org/10.15252/msb.20188503.
28. Lin, Y.-H., Platt, M.P., Fu, H., Gui, Y., Wang, Y., Gonzalez-Juarbe, N., Zhou, D., and Yu, Y. (2020). Global Proteome and Phosphoproteome Characterization of Sepsis-induced Kidney Injury. Mol. Cell. Proteomics *19*, 2030–2047. https://doi.org/10.1074/mcp.RA120.002235.
29. Lin, Y.-H., Eguez, R.V., Torralba, M.G., Singh, H., Golusinski, P., Golusinski, W., Masternak, M., Nelson, K.E., Freire, M., and Yu, Y. (2019). Self-Assembled STrap for Global Proteomics and Salivary Biomarker Discovery. J. Proteome Res. *18*, 1907–1915. https://doi.org/10.1021/acs.jproteome.9b00037.
30. Sielaff, M., Kuharev, J., Bohn, T., Hahlbrock, J., Bopp, T., Tenzer, S., and Distler, U. (2017). Evaluation of FASP, SP3, and iST Protocols for Proteomic Sample Preparation in the Low Microgram Range. J. Proteome Res. *16*, 4060–4072. https://doi.org/10.1021/acs.jproteome.7b00433.
31. Hatano, A., Takami, T., and Matsumoto, M. (2023). In situ digestion of alcohol-fixed cells for quantitative proteomics. J. Biochem. *173*, 243–254. https://doi.org/10.1093/jb/mvac101.
32. Kelly, V., Al-Rawi, A., Lewis, D., Kustatscher, G., and Ly, T. (2022). Low Cell Number Proteomic Analysis Using In-Cell Protease Digests Reveals a Robust Signature for Cell Cycle State Classification. Mol. Cell. Proteomics *21*, 100169. https://doi.org/10.1016/j.mcpro.2021.100169.
33. Hoque, P., Romero, B., Akins, R.E., and Batish, M. (2023). Exploring the Multifaceted Biologically Relevant Roles of circRNAs: From Regulation, Translation to Biomarkers. Cells *12*, 2813. https://doi.org/10.3390/cells12242813.
34. Butter, F., Scheibe, M., Mörl, M., and Mann, M. (2009). Unbiased RNA-protein interaction screen by quantitative proteomics. Proc. Natl. Acad. Sci. USA *106*, 10626–10631. https://doi.org/10.1073/pnas.0812099106.
35. Yang, J.-H., Pandey, P.R., and Gorospe, M. (2021). Identification of circRNA-Interacting Proteins by Affinity Pulldown. Methods Mol. Biol. *2372*, 193–202. https://doi.org/10.1007/978-1-0716-1697-0_17.
36. Huang, A., Zheng, H., Wu, Z., Chen, M., and Huang, Y. (2020). Circular RNA-protein interactions: functions, mechanisms, and identification. Theranostics *10*, 3503–3517. https://doi.org/10.7150/thno.42174.
37. Huang, S., Li, X., Zheng, H., Si, X., Li, B., Wei, G., Li, C., Chen, Y., Chen, Y., Liao, W., et al. (2019). Loss of Super-Enhancer-Regulated circRNA Nfix Induces Cardiac Regeneration After Myocardial Infarction in Adult Mice. Circulation *139*, 2857–2876. https://doi.org/10.1161/CIRCULATIONAHA.118.038361.
38. Yi, W., Li, J., Zhu, X., Wang, X., Fan, L., Sun, W., Liao, L., Zhang, J., Li, X., Ye, J., et al. (2020). CRISPR-assisted detection of RNA-protein interactions in living cells. Nat. Methods *17*, 685–688. https://doi.org/10.1038/s41592-020-0866-0.
39. Kwon, E., Todorova, K., Wang, J., Horos, R., Lee, K.K., Neel, V.A., Negri, G.L., Sorensen, P.H., Lee, S.W., Hentze, M.W., and Mandinova, A. (2018). The RNA-binding protein YBX1 regulates epidermal progenitors at a post-transcriptional level. Nat. Commun. *9*, 1734. https://doi.org/10.1038/s41467-018-04092-0.
40. Wu, R., Cao, S., Li, F., Feng, S., Shu, G., Wang, L., Gao, P., Zhu, X., Zhu, C., Wang, S., and Jiang, Q. (2022). RNA-binding protein YBX1 promotes brown adipogenesis and thermogenesis via PINK1/PRKN-mediated mitophagy. FASEB J *36*, e22219. https://doi.org/10.1096/fj.202101810RR.
41. Li, H., Zhang, D., Fu, Q., Wang, S., Wang, Z., Zhang, X., Chen, X., Zhu, X., An, N., Chen, Y., et al. (2023). YBX1 as an oncogenic factor in T-cell acute lymphoblastic leukemia. Blood Adv. *7*, 4874–4885. https://doi.org/10.1182/bloodadvances.2022009648.
42. Koppula, A., Abdelgawad, A., Guarnerio, J., Batish, M., and Parashar, V. (2022). CircFISH: A Novel Method for the Simultaneous Imaging of Linear and Circular RNAs. Cancers *14*, 428. https://doi.org/10.3390/cancers14020428.
43. Qadir, J., Li, F., and Yang, B.B. (2022). Circular RNAs modulate Hippo-YAP signaling: functional mechanisms in cancer. Theranostics *12*, 4269–4287. https://doi.org/10.7150/thno.71708.
44. Radanova, M., Mihaylova, G., Nazifova-Tasinova, N., Levkova, M., Tasinov, O., Ivanova, D., Mihaylova, Z., and Donev, I. (2021). Oncogenic Functions and Clinical Significance of Circular RNAs in Colorectal Cancer. Cancers *13*, 3395. https://doi.org/10.3390/cancers13143395.
45. Sim, S., Yao, J., Weinberg, D.E., Niessen, S., Yates, J.R., 3rd, and Wolin, S.L. (2012). The zipcode-binding protein ZBP1 influences the subcellular location of the Ro 60-kDa autoantigen and the noncoding Y3 RNA. RNA *18*, 100–110. https://doi.org/10.1261/rna.029207.111.
46. Hedrich, S., Schlömann, M., and Johnson, D.B. (2011). The iron-oxidizing proteobacteria. Microbiology (Read.) *157*, 1551–1564. https://doi.org/10.1099/mic.0.045344-0.
47. Weiss, J.V., Emerson, D., Backer, S.M., and Megonigal, J.P. (2003). Enumeration of Fe(II)-oxidizing and Fe(III)-reducing bacteria in the root zone of wetland plants: Implications for a rhizosphere iron cycle. Biogeochemistry *64*, 77–96. https://doi.org/10.1023/A:1024953027726.
48. Chan, C.S., Dykes, G.E., Hoover, R.L., Limmer, M.A., and Seyfferth, A.L. (2023). Gallionellaceae in rice root plaque: metabolic roles in iron oxidation, nutrient cycling, and plant interactions. Appl. Environ. Microbiol. *89*, e0057023. https://doi.org/10.1128/aem.00570-23.
49. Emerson, D., and Moyer, C. (1997). Isolation and characterization of novel iron-oxidizing bacteria that grow at circumneutral pH. Appl. Environ. Microbiol. *63*, 4784–4792. https://doi.org/10.1128/aem.63.12.4784-4792.1997.
50. Emerson, D., Field, E.K., Chertkov, O., Davenport, K.W., Goodwin, L., Munk, C., Nolan, M., and Woyke, T. (2013). Comparative genomics of freshwater Fe-oxidizing bacteria: implications for physiology, ecology, and systematics. Front. Microbiol. *4*, 254. https://doi.org/10.3389/fmicb.2013.00254.
51. Zhou, N., Keffer, J.L., Polson, S.W., and Chan, C.S. (2022). Unraveling Fe(II)-Oxidizing Mechanisms in a Facultative Fe(II) Oxidizer, Sideroxydans lithotrophicus Strain ES-1, via Culturing, Transcriptomics, and Reverse Transcription-Quantitative PCR. Appl. Environ. Microbiol. *88*, e0159521. https://doi.org/10.1128/AEM.01595-21.
52. Zhou, N., Kupper, R.J., Catalano, J.G., Thompson, A., and Chan, C.S. (2022). Biological Oxidation of Fe(II)-Bearing Smectite by Microaerophilic Iron Oxidizer Sideroxydans lithotrophicus Using Dual Mto and Cyc2 Iron Oxidation Pathways. Environ. Sci. Technol. *56*, 17443–17453. https://doi.org/10.1021/acs.est.2c05142.
53. Nagaraj, N., Wisniewski, J.R., Geiger, T., Cox, J., Kircher, M., Kelso, J., Pääbo, S., and Mann, M. (2011). Deep proteome and transcriptome mapping of a human cancer cell line. Mol. Syst. Biol. *7*, 548. https://doi.org/10.1038/msb.2011.81.
54. Cooper, R.E., Wegner, C.-E., Kügler, S., Poulin, R.X., Ueberschaar, N., Wurlitzer, J.D., Stettin, D., Wichard, T., Pohnert, G., and Küsel, K. (2020). Iron is not everything: unexpected complex metabolic responses

between iron-cycling microorganisms. ISME J. *14*, 2675–2690. https://doi.org/10.1038/s41396-020-0718-z.

55. Beckwith, C.R., Edwards, M.J., Lawes, M., Shi, L., Butt, J.N., Richardson, D.J., and Clarke, T.A. (2015). Characterization of MtoD from Sideroxydans lithotrophicus: a cytochrome c electron shuttle used in lithoautotrophic growth. Front. Microbiol. *6*, 332. https://doi.org/10.3389/fmicb.2015.00332.

56. HaileMariam, M., Eguez, R.V., Singh, H., Bekele, S., Ameni, G., Pieper, R., and Yu, Y. (2018). S-Trap, an Ultrafast Sample-Preparation Approach for Shotgun Proteomics. J. Proteome Res. *17*, 2917–2924. https://doi.org/10.1021/acs.jproteome.8b00505.

57. Macklin, A., Khan, S., and Kislinger, T. (2020). Recent advances in mass spectrometry based clinical proteomics: applications to cancer research. Clin. Proteomics *17*, 17. https://doi.org/10.1186/s12014-020-09283-w.

58. Cong, Y., Motamedchaboki, K., Misal, S.A., Liang, Y., Guise, A.J., Truong, T., Huguet, R., Plowey, E.D., Zhu, Y., Lopez-Ferrer, D., and Kelly, R.T. (2020). Ultrasensitive single-cell proteomics workflow identifies >1000 protein groups per mammalian cell. Chem. Sci. *12*, 1001–1006. https://doi.org/10.1039/D0SC03636F.

59. Zhu, Y., Piehowski, P.D., Zhao, R., Chen, J., Shen, Y., Moore, R.J., Shukla, A.K., Petyuk, V.A., Campbell-Thompson, M., Mathews, C.E., et al. (2018). Nanodroplet processing platform for deep and quantitative proteome profiling of 10–100 mammalian cells. Nat. Commun. *9*, 882. https://doi.org/10.1038/s41467-018-03367-w.

60. Kassem, S., van der Pan, K., de Jager, A.L., Naber, B.A.E., de Laat, I.F., Louis, A., van Dongen, J.J.M., Teodosio, C., and Díez, P. (2021). Proteomics for Low Cell Numbers: How to Optimize the Sample Preparation Workflow for Mass Spectrometry Analysis. J. Proteome Res. *20*, 4217–4230. https://doi.org/10.1021/acs.jproteome.1c00321.

61. Kostas, J.C., Greguš, M., Schejbal, J., Ray, S., and Ivanov, A.R. (2021). Simple and Efficient Microsolid-Phase Extraction Tip-Based Sample Preparation Workflow to Enable Sensitive Proteomic Profiling of Limited Samples (200 to 10,000 Cells). J. Proteome Res. *20*, 1676–1688. https://doi.org/10.1021/acs.jproteome.0c00890.

62. Kelly, R.T. (2020). Single-cell Proteomics: Progress and Prospects. Mol. Cell. Proteomics *19*, 1739–1748. https://doi.org/10.1074/mcp.R120.002234.

63. Webber, K.G.I., Huang, S., Truong, T., Heninger, J.L., Gregus, M., Ivanov, A.R., and Kelly, R.T. (2024). Open-tubular trap columns: towards simple and robust liquid chromatography separations for single-cell proteomics. Mol. Omics *20*, 184–191. https://doi.org/10.1039/D3MO00249G.

64. Johnston, S.M., Webber, K.G.I., Xie, X., Truong, T., Nydegger, A., Lin, H.-J.L., Nwosu, A., Zhu, Y., and Kelly, R.T. (2023). Rapid, One-Step Sample Processing for Label-Free Single-Cell Proteomics. J. Am. Soc. Mass Spectrom. *34*, 1701–1707. https://doi.org/10.1021/jasms.3c00159.

65. Ctortecka, C., Clark, N.M., Boyle, B., Seth, A., Mani, D.R., Udeshi, N.D., and Carr, S.A. (2024). Automated single-cell proteomics providing sufficient proteome depth to study complex biology beyond cell type classifications. Preprint at bioRxiv. https://doi.org/10.1101/2024.01.20.576369.

66. Sanchez-Avila, X., Truong, T., Xie, X., Webber, K.G.I., Johnston, S.M., Lin, H.-J.L., Axtell, N.B., Puig-Sanvicens, V., and Kelly, R.T. (2023). Easy and Accessible Workflow for Label-Free Single-Cell Proteomics. J. Am. Soc. Mass Spectrom. *34*, 2374–2380. https://doi.org/10.1021/jasms.3c00240.

67. Guzman, U.H., Martinez-Val, A., Ye, Z., Damoc, E., Arrey, T.N., Pashkova, A., Renuse, S., Denisov, E., Petzoldt, J., Peterson, A.C., et al. (2024). Ultra-fast label-free quantification and comprehensive proteome coverage with narrow-window data-independent acquisition. Nat. Biotechnol. https://doi.org/10.1038/s41587-023-02099-7.

68. Ye, Z., Sabatier, P., Martin-Gonzalez, J., Eguchi, A., Lechner, M., Østergaard, O., Xie, J., Guo, Y., Schultz, L., Truffer, R., et al. (2024). One-Tip enables comprehensive proteome coverage in minimal cells and single zygotes. Nat. Commun. *15*, 2474. https://doi.org/10.1038/s41467-024-46777-9.

69. Specht, H., Emmott, E., Petelski, A.A., Huffman, R.G., Perlman, D.H., Serra, M., Kharchenko, P., Koller, A., and Slavov, N. (2021). Single-cell proteomic and transcriptomic analysis of macrophage heterogeneity using SCoPE2. Genome Biol. *22*, 50. https://doi.org/10.1186/s13059-021-02267-5.

70. Brunner, A.-D., Thielert, M., Vasilopoulou, C., Ammar, C., Coscia, F., Mund, A., Hoerning, O.B., Bache, N., Apalategui, A., Lubeck, M., et al. (2022). Ultra-high sensitivity mass spectrometry quantifies single-cell proteome changes upon perturbation. Mol. Syst. Biol. *18*, e10798. https://doi.org/10.15252/msb.202110798.

71. Peters-Clarke, T.M., Liang, Y., Mertz, K.L., Lee, K.W., Westphall, M.S., Hinkle, J.D., McAlister, G.C., Syka, J.E.P., Kelly, R.T., and Coon, J.J. (2024). Boosting the Sensitivity of Quantitative Single-Cell Proteomics with Activated Ion-Tandem Mass Tags (AI-TMT). Preprint at bioRxiv. https://doi.org/10.1101/2024.02.24.581874.

72. Yu, Y., and Pieper, R. (2019). Using Proteomics to Identify Inflammation During Urinary Tract Infection. In Proteus mirabilis: Methods and Protocols, M.M. Pearson, ed. (Springer), pp. 259–272. https://doi.org/10.1007/978-1-4939-9601-8_22.

73. Yu, Y., Suh, M.-J., Sikorski, P., Kwon, K., Nelson, K.E., and Pieper, R. (2014). Urine Sample Preparation in 96-Well Filter Plates for Quantitative Clinical Proteomics. Anal. Chem. *86*, 5470–5477. https://doi.org/10.1021/ac5008317.

74. Batish, M., van den Bogaard, P., Kramer, F.R., and Tyagi, S. (2012). Neuronal mRNAs travel singly into dendrites. Proc. Natl. Acad. Sci. USA *109*, 4645–4650. https://doi.org/10.1073/pnas.1111226109.

75. Tyanova, S., Temu, T., and Cox, J. (2016). The MaxQuant computational platform for mass spectrometry-based shotgun proteomics. Nat. Protoc. *11*, 2301–2319. https://doi.org/10.1038/nprot.2016.136.

STAR★METHODS

KEY RESOURCES TABLE

REAGENT or RESOURCE	SOURCE	IDENTIFIER
Antibodies		
Goat polyclonal anti-rabbit IgG	Invitrogen	Cat# F-2765
Rabbit polyclonal anti-YBX-1	Cell Signaling Technology	Cat# D299
Bacterial and virus strains		
E. coli BL21(DE3) cells	Agilent	Cat# 200131
Biological samples		
HEK293	ATTC	Cat# CRL-1573
Jurkat, Clone E6-1	ATTC	Cat# TIB-152
Yeast cells	Horizon Discovery	Cat# YSC3867
Chemicals, peptides, and recombinant proteins		
Acetone, HPLC grade	Fisher	Cat# A949
Acetic acid, LC/MS grade	Fisher	Cat# A11350
Acetonitrile, LC/MS grade	Honeywell Chemicals	Cat# 349674
Ammonium bicarbonate	Acros Organics	Cat# 393212500
Ammonium chloride	Fisher	Cat# A661
Biotin	Sigma-Aldrich	Cat# B4501
Calcium chloride dihydrate	VWR	Cat# BDH0224
Chloroacetamide	Thermo Scientific	Cat# AAA1523830
DAPI	Sigma-Aldrich	Cat# D9542
Dulbecco’s Modified Eagle medium	Life Technologies	Cat# 11-965-092
Dynabeads MyOne Streptavidin C1	Invitrogen	Cat# 65002
Ethanol	Decon Labs	Cat# 2701
Fetal bovine serum	Gibco	Cat# 16000044
Formaldehyde	Sigma-Aldrich	Cat# F8775
Formic acid, LC/MS grade	Fisher	Cat# A117
Lenti-X concentrator	Takara Bio	Cat# 631231
L-glutamine	Gibco	Cat# 25030081
Magnesium sulfate heptahydrate	Acros Organics	Cat# 423905000
Methanol, LC/MS grade	Fisher	Cat# A456
Penicillin/streptomycin	Sigma-Aldrich	Cat# P4458
Phosphate buffered saline, 10x	Fisher	Cat# BP399
Phosphoric acid, HPLC grade	Honeywell	Cat# 79606
Potassium phosphate dibasic anhydrous	Fisher	Cat# P288
Puromycin dihydrochloride	Sigma-Aldrich	Cat# P9620
RIPA buffer	Millipore	Cat# 20–188
RPMI 1640	Corning	Cat# 10-041-CV
SD-Ura medium	Fisher	Cat# MP114813065
Sodium borohydride	Sigma-Aldrich	Cat# 213462
Sodium dodecyl sulfate, 10% solution	Fisher	Cat# BP2436
Sodium thiosulfate pentahydrate	Fisher	Cat# S445
SYTO 13	Invitrogen	Cat# S7575
Triethylammonium bicarbonate buffer	Fisher	Cat# 60-044-973
Trifluoroacetic acid, 99%	Acros Organics	Cat# 13972–5000
Tris-HCl Buffer, pH 8.0	Fisher	Cat# 15-568-025
Tris(2-carboxyethyl)phosphine	Fisher	Cat# AA4058704

(*Continued on next page*)

Continued

REAGENT or RESOURCE	SOURCE	IDENTIFIER
Trypsin, MS Grade	Promega	Cat# V5111
Urea	Acros Organics	Cat# 1407500010
Water, LC/MS grade	Fisher	Cat# W64
Wolfe's vitamin supplement	ATCC	MD-VS
Deposited data		
Mass spectrometry raw data	This study	MassIVE: MSV000092423 and MSV000094082
Experimental models: Organisms/strains		
Sideroxydans lithotrophicus ES-1	David Emerson (Bigelow Laboratory for Ocean Sciences)	ES-1
Oligonucleotides		
CircNFIX gRNA targeting sequence: TTGTCCACACTCCGGGATGAGTT	This paper	N/A
Linear NFIX gRNA targeting sequence: GGGGAGAAGAAATTTTGAGAATG	This paper	N/A
Sm-FISH probes: See Table S1	This paper	N/A
Recombinant DNA		
CARPID BASU-dCasRx	Yi et al.[38]	Addgene Plasmid #153209
pLentiRNAGuide_001 - hU6-RfxCas13d-DR1-BsmBI-EFS-Puro-WPRE	Yi et al.[38]	Addgene Plasmid #138150
Software and algorithms		
MaxQuant version 1.6.3.4	N/A	https://www.maxquant.org/maxquant/
Perseus version 1.6.2.3	N/A	https://www.maxquant.org/perseus/
MetaMorph v7.8.13.0	Molecular Devices	https://www.moleculardevices.com/
GraphPad Prism version 9.0	GraphPad	https://www.graphpad.com/
MATLAB version 2021b	MathWorks	https://www.mathworks.com/
Other		
Empore C18 StageTips	Fisher	Cat# 13-110-055
Empore E3tip	Fisher	Cat#13-110-092
Empore E3filter	Fisher	Cat# 13-110-084
Empore E3cardridge	Fisher	Cat# 13-110-079
Empore E3plate	Fisher	Cat# 13-110-077
Empore E4tip	Fisher	Cat# 13-110-088
Empore E4cardridge	CDS Analytical	https://www.cdsanalytical.com/e3technology
Glass beads, 20–50 μm	NIST	SRM 1003c
Glass beads, 9–13 μm	Sigma-Aldrich	Cat# 440345
Microcon-30kDa centrifugal filters	Millipore	Cat# MRCF0R030
PepMap100 C18 analytical column	Thermo Scientific	Cat# 164570
PepMap100 C18 trap column	Thermo Scientific	Cat# 174500
Whatman GF/F glass microfiber filters, 0.7 μm	Cytiva	Cat# 1825–090

RESOURCE AVAILABILITY

Lead contact

Further information and requests for resources and reagents should be directed to and will be fulfilled by the lead contact, Yanbao Yu (yybyu@udel.edu).

Materials availability

- This study did not generate new plasmids or cell lines.

- This study generated new filter devices for sample preparation. E3filter (spin column, 0.5 mL), E3tip (pipette tips, 10 and 200 μL), E3plate (96-well plate, 1.2 mL), and E3cartridge (cartridge, 3 mL), as well as E4 devices (tips , filters, cartridges, and plates) are available from CDS Analytical in the listed current formats at https://www.cdsanalytical.com/e3technology.
- Please refer to "key resources table" for more details.

Data and code availability

- The MS raw files associated with this study have been deposited to the MassIVE server (https://massive.ucsd.edu/) with the dataset identifiers MSV000092423 and MSV000094082 and are publicly available as of the date of publication. Accession numbers are also listed in the key resources table.
- This study does not report original code.
- Any additional information required to reanalyze the data reported in this paper is available from the lead contact upon request.

EXPERIMENTAL MODEL AND STUDY PARTICIPANT DETAILS

The collection of saliva samples was approved under a Biological Material Registration Form, and collection from a single individual was not considered generalizable research that would require University of Delaware Institutional Review Board approval and oversight.

METHOD DETAILS

Cell culture, and tissue and specimen collection

For mammalian cell culture (HEK293 and Jurkat), all the culturing reagents were purchased from Corning, and cell lines were obtained from American Type Culture Collection (ATTC, Manassas, VA), unless otherwise indicated. For HEK293 cells, the cell line was thawed from a P1 stock and cultured in Dulbecco's modified eagle medium (DMEM), 10% fetal bovine serum (FBS) (Atlantic Biologicals), 20 mM L-glutamine, 1% penicillin-streptomycin, on a 100-mm dish and grown in a humidified incubator at 37°C and 5% CO_2. After 3–4 days after thawing, the cell line was tested negative for mycoplasma. Cells were harvested when reaching 90% confluence and washed twice with cold 1x PBS. Cell pellets were stored at −80°C until further use. Jurkat cells were purchased from ATCC (Jurkat, Clone E6-1), and the passage number is P9. The culturing medium included RPMI 1640 (Cat# 10041CV; Corning), 10% FBS (Cat# 16000044; Gibco) and 1% Penstrep (Cat# 15070063; Gibco). The cells were incubated at 37°C in a CO_2 Incubator (Heracell 150i, 150L, Thermo Scientific). Cells were examined and counted using trypan blue staining and the Countess 3 FL automated cell counter (Cat# AMQAF2000, ThermoFisher Scientific).

For *E. coli* cell culture, all the procedures were performed close to a Bunsen burner flame and all the media was sterilized by autoclaving. Other reagents were purchased from Sigma-Aldrich, unless otherwise indicated. The *E. coli* strain BL21(DE3) was obtained from Agilent Technologies (Santa Clara, CA). The cells were streaked out on a lysogeny broth (LB) agar plate and incubated overnight at 37°C. The next day, a single colony was picked and incubated in a 3 mL LB and incubated overnight at 37°C in a shaking incubator. The next morning, 100 mL of LB was inoculated with 1 mL of the overnight culture and grown until OD_{600} = 0.8. Cells were pelleted at 4,000 rpm for 5 min and washed twice with 1x PBS. Cell pellets were stored at −80°C until further use.

For yeast cell culture, the yeast strain was purchased from Horizon (Horizon, Cat. #YSC3867). Cells were grown in SD-Ura media (6.7 g/L yeast nitrogen base without amino acids, 0.77 g/L-Ura DO supplement, 3% glycerol. 2% dextrose) at 30°C to an OD_{600} of around 1.2. The cells were centrifuged at 500 x g for 5 min, washed with cold PBS, and then collected for immediate in-cell digestion (as described below).

Saliva specimen was collected from a healthy donor following a procedure described previously.[29] Briefly, the donor was asked to not eat or drink for at least 1 h before saliva collection. A sample was obtained by draining the saliva from the mouth directly into a 15-mL falcon tube. Up to 5 mL of unstimulated whole saliva was collected between 10 a.m. and 12 p.m. Immediately after collection, the sample was deactivated with SDS buffer (4% SDS, 100 mM Tris-HCl, pH 8.0) and boiled at 95°C for 10 min. Afterward, the sample was centrifuged at 14,000 x g for 20 min and the supernatant was collected for proteomics analysis.

E3filter experiments and SP4-GB, FASP digestion of *E. coli* proteins

For the initial experiments, the glass beads (GBs) were purchased from Sigma (Cat# 440345, 9–13 μm; NIST1003C, 20–50 μm). The E3filters were assembled following a procedure reported previously.[24] In brief, the Microcon filter device (Cat# MRCF0R030; MilliporeSigma) was first disassembled to discard the pre-installed membrane disc. Two new discs of GB membrane were cut using a 10''/32 hole punch (McGill 2'' Reach Punchline Hole Punch), and then reassembled into the device. Alternatively, two other types of filters could be used, one from Thomson Instrument (Cat# 35530) and the other from Epoch Life Science (Cat# 1920), both of which worked well in our experiments. Please be kindly noted that, during the revision process of the manuscript, the E3filters have become commercially available from CDS Analytical (Cat# 70-2019-3101-0; https://www.cdsanalytical.com/e3technology). Please find step-by-step protocols from the Methods S1. The E3filters were first rinsed with 80% acetonitrile and then 50 mM triethylammonium bicarbonate (TEAB) by centrifuging at 4,000 rpm for 1–2 min. To perform protein reduction and alkylation, around 20 μg aliquot of *E. coli* lysate in SDS lysis buffer (5% SDS, 100 mM Tris HCl, pH = 8.0) was mixed with 50 mM TEAB, 10 mM Tris(2-carboxyethyl)phosphine

(TCEP), and 40mM chloroacetamide (CAA), incubated at 70°C for 30 min with gentle shaking (400 rpm). Afterward, the proteins were mixed with 4x volume of 80% acetonitrile, and transferred to E3filters. After centrifuging for 1-2 min at 4,000 rpm to discard the flow through, the proteins on the filter were washed with 80% ethanol for three times. For protein digestion, the E3filters were transferred to clean collection tubes after adding 200 μL of TEAB (50mM) and 0.4 μg of trypsin. The samples were incubated at 37°C with gentle shaking (400 rpm) for 16–18 h. To elute peptides, sequential elution with 50mM TEAB, and 50% acetonitrile and 0.1% (v/v) formic acid in water (200 μL each) were performed by centrifuging at 4,000 rpm for 1-2min. The elution was pooled together, dried by SpeedVac, and subjected to C18-based StageTip desalting as we described before.[72] For the E3filter experiment described here, and all the other digestion experiments described below, at least three replicates were performed. Please be aware that these digestion procedures shown here (and below) were utilized only in the initial experiments. Optimized and latested protocols can be found in Methods S1.

For the SP4-GB experiment, the 10-μm GBs were purchased from Sigma (Cat# 440345). The beads were then processed as Johnston et al. described before.[14] After sequential washes, the pelleted beads were resuspended into acetonitrile to obtain around 12.5 mg/mL concentration. Similar to E3filter procedures, around 20 μg of *E. coli* proteins after reduction and alkylation was added to GB solution along with 4x volume of 80% acetonitrile. The samples were centrifuged at maximum speed (13,000 rpm) for 2 min. The supernatant was discarded, and the pellet was washed with 80% ethanol for three times. For protein digestion, 200 μL of TEAB (50mM) and 0.4 μg of trypsin were added to the samples, which were incubated at 37°C with gentle shaking (400 rpm) for 16–18 h. To collect peptides, sequential elution similar to E3filters were performed. The elution was dried by SpeedVac, and desalted as described above.

For the FASP experiment, the Microcon 30-kDa cutoff filter devices were purchased from Millipore (Cat# MRCF0R030; Millipore). Similar to SP4-GB procedures, the 20 μg of *E. coli* proteins after reduction and alkylation was mixed with 200 μL 8M urea buffer, transferred to filter device, and centrifuged at 14,000 x g for 15min. After discarding the flow through, the proteins were washed again with the urea buffer two more times, and with 50mM ammonium bicarbonate (ABC) two more times. The proteins were digested with 0.4 μg of trypsin in 200 μL ABC buffer, incubated at 37°C with gentle shaking (400 rpm) for 16–18 h. To collect peptides, 200 μL ABC buffer was added, centrifuged at 14,000 x g for 10-15min. The flow through was transferred to a clean tube. This step was repeated two more times. The elution was pooled, dried in SpeedVac, and desalted as described above.

E3tip experiments and FA-SPEED digestion of HEK293 proteins

The E3tips were packed following a protocol similar to the preparation of multi-disk StageTips.[23] Briefly, the GB membrane disk was prepared with four punches of the GB membrane using a 16-gauge blunt end needle, and installed into 200 μL pipette tips with firm tightness. Here, we noticed that stacking four membranes together with one punch can give us a better-quality packing than punching four times from a single membrane. After packing, the tips were first rinsed with 80% acetonitrile, and then 50mM TEAB before sample loading. Around $5x10^5$ HEK293 cell pellets were mixed with 4x volume of pure tri trifluoroacetic acid (TFA), gently mixed and incubated at room temperature for 3–5 min. Protein concentration was estimated by running aliquots of the lysate onto SDS PAGE along with known concentration of bovine serum albumin (BSA) as we described previously.[73] Around 10 μg of proteins were aliquoted, neutralized with 10x volume 2 M tris base buffer, and reduced/alkylated by mixing with 10 mM TCEP and 40 mM CAA. The proteins were precipitated with 4x volume of cold acetone, and incubate for 5 min. The precipitates were then transferred to E3tips and centrifuged at 2,000 rpm for 1-2 min to discard the flow through. The tips were washed with 200 μL 80% acetone for three times by centrifuging at 2,000 rpm for 1–2 min and discarding the flow through, respectively. Here, depending on the amount of total proteins loaded onto the tips, centrifugation speed may vary (e.g., going up to 5,000 rpm if more materials were loaded. Recommended loading capacity for E3tips is 20 μg or less). Afterward, the tips were transferred to new collection tubes. For protein digestion, 150 μL digestion buffer (50 mM TEAB) and 0.2 μg of trypsin were added followed by incubation at 37°C for 16–18 h. To elute peptides, 200 μL of 0.1% formic acid in 50% acetonitrile was added and centrifuged at 2,000 rpm for 1–2 min. This step repeated two additional times. The elution was pooled, dried in SpeedVac, and desalted using C18 StageTips (CDS Analytical) as described above.

For FA-SPEED experiment, PTFE filters with 0.2 μm pore size were purchased from Thomson Solutions (Cat# 34430). Similar to the experiment above, aliquots of 10 μg of proteins were pretreated, and then precipitated with cold acetone. Instead of loading onto E3tips, the precipitates were loaded onto Thomson filters. The filters were centrifuged at 4,000 rpm for 1–2 to discard flow through followed by washing with 80% acetone for three times. As a note, because the Thomson filters are not designed for centrifugation, so the associated collection tube from the manufacture does not work. We took regular 2.0 mL microtubes (for instance, Axygen MaxyClear Snaplock Microtubes, 2.0 mL, Cat# MCT-200-L-C; or Thermo Scientific Low Protein Binding Microcentrifuge Tubes, Cat# 88379) as collection tubes for Thomson filters as showed before.[24] We poked a hole at around the 0.8-mL mark as a vent. Afterward, the proteins were digested, and the resulting peptides were desalted the same as described above.

E3tip experiments and S-Tip kidney protein digestion

The E3tips were packed as described above. The mouse kidney lysate was adopted from one of our previous studies.[28] Around 20 μg of proteins in SDS lysis buffer (4% SDS, 100 mM Tris HCl, pH 8.0) was aliquoted, reduced/alkylated with 10 mM TCEP and 40mM CAA, and incubated at 95°C for 10 min. The rest of the procedures were following the suspension trap protocol described before.[8] Briefly, phosphoric acid (with protein volume ratio 10:1) was added to acidify the solution followed by precipitation with 6x volume of 90% methanol in 50 mM TEAB. The protein precipitates were transferred to E3tips, and centrifuged at 2,000 rpm for 1-2min to

discard the flow through. Two to three additional wash steps were performed with 200 μL of 90% methanol in 50 mM TEAB solution each. Afterward, the E3tips were transferred to clean collection tubes. For protein digestion, 150 μL digestion buffer (50 mM TEAB) and 0.4 μg of trypsin (protein:enzyme ratio = 50:1) were added followed by incubation at 37°C for 16–18 h. To elute peptides, three sequential washes with 200 μL of 50 mM TEAB, 0.2% formic acid water, 0.2% formic acid in 50% acetonitrile/50% water were performed, respectively. The elution was pooled, dried by SpeedVac, and desalted using C18 StageTips (CDS Analytical) as described above.

The S-Tips were packed by similarly to E3tips, but with glass fiber membranes. We obtained the Whatman GF/F membrane (0.7 μm pore size) from Cytiva, and 0.2 μm pore size glass fiber membrane from Graver Technologies (Glasgow, DE). Two layers of each were cut and packed into the 200 μL pipette tips (0.7 μm membrane on top, and 0.2 μm at the bottom). The procedures to process the proteins and peptides were the same as described above.

E3cartridge and E3plate saliva experiments

Saliva specimen was collected from a healthy donor following a procedure described previously.[29] Briefly, the donor was asked to not eat or drink for at least 1 h before saliva collection. A sample was obtained by draining the saliva from the mouth directly into a 15-mL falcon tube. Up to 5 mL of unstimulated whole saliva was collected between 10 a.m. and 12 p.m. Immediately after collection, the sample was deactivated with SDS buffer (4% SDS, 20 mM DTT, 100 mM Tris-HCl, pH 8.0) and boiled at 95°C for 10 min. Afterward, the sample was centrifuged at 14,000 x g for 20 min and the supernatant was collected for proteomics analysis. Aliquots of 100 μL of the supernatant were first alkylated with 50 mM iodoacetamide by incubation at room temperature in the dark for around 30 min, then precipitated with 80% acetonitrile. The precipitates were transferred to E3cartridges and E3plates that were precast with GB membranes and processed similarly to SP4-GB method. In brief, the cartridges and plates were rinsed sequentially with 0.5 mL of 80% acetonitrile and 50 mM TEAB. The liquid transferring from cartridges was done manually using a 10-mL syringe, whereas centrifugation (4,000 rpm for 1–2 min) was used for the plates. A clean deep-well plate was used to collect the waste. After sample loading, two wash steps were performed with 80% ethanol. For digestion, 0.1 μg trypsin was added with 200 μL TEAB solution (50 mM) to the filters followed by overnight incubation at 37°C with gentle shaking. The peptides were collected by sequential elution with 50mM TEAB, and 50% acetonitrile and 0.1% formic acid in water (200 μL each). The elution was pooled, dried, and cleaned using C18 StageTips (CDS Analytical) as described above.

E4tip and on-filter in-cell (OFIC) digestion experiments

E4tips were packed similarly to E3tips but with a GB|C18 membrane. Aliquots of freshly collected Jurkat or yeast cells were mixed with 200 μL of pure methanol by pipetting up and down several times, and then transferred to E4tips followed by centrifuging at 4,000 rpm for 2 min to discard the flow through. Another 200 μL of methanol was added to the E4tips, which were then incubated at 4°C for at least 30 min. The E4tips were centrifuged again followed by reduction and alkylation with 10 mM TCEP and 40 mM CAA in 100 μL of 50 mM TEAB and incubation at 70°C for around 30 min. The E4tips were centrifuged and washed two times with 200 μL of 50 mM TEAB. To digest proteins in the fixed cells, the protein quantity was first estimated based on an assumption that each cell contains around 200 pg of proteins.[10] Then, trypsin was added based on the enzyme-to-protein ratio of 1:50. After overnight incubation at 37°C with gentle shaking, the E4tips were first acidified with 1% formic acid (final concentration). After centrifugation to discard the flow-through, the E4tips were wash with 200 μL of 0.5% acetic acid in water. The desalted peptides were eluted into clean collection tubes with sequential elution of 60% acetonitrile and 0.5% acetic acid (once), and 80% acetonitrile and 0.5% acetic acid (twice). The elution was pooled, dried, and stored in −80°C until LC-MS/MS analysis.

Alternatively, instead of doing reduction and alkylation on protein level, we also tried both reactions on peptide level. Basically, after digestion 10 mM TCEP and 40 mM CAA (final concentrations) were added to the E4tips followed by incubation at 70°C for around 30min. Afterward, the tips were acidified, washed, and eluted similarly to the procedures described above. Detailed protocols could be found from Methods S1.

Sideroxydans lithotrophicus ES-1 cell culture, collection, and OFIC sample preparation

Sideroxydans lithotrophicus ES-1 was grown in modified Wolfe's minimal medium (MWMM) plus trace minerals and vitamins, buffered with 20 mM MES pH 6.0 with a one-time addition of 10 mM thiosulfate as the electron donor and a daily flushing with 2% oxygen (in 20% carbon dioxide/78% nitrogen) as the electron acceptor as described previously.[51] The cell number was determined by counting Syto13-stained cells under fluorescent microscopy using a Hausser counting chamber. ES-1 was collected at stationary phase by filtering through the E4cartridges. The cells were rinsed twice by adding 0.5 mL of cold PBS and centrifuging at 500 x g for 1–2 min, and then were fixed by adding 500 μL of pure methanol followed by incubation at 4°C for 30 min. The cells were then processed following the E4technology OFIC digestion protocol as described above.

LC-MS/MS analysis

Peptides were separated on an Ultimate 3000 RSLCnano system coupled with a trap column (PepMap100 C18, 300 μm × 2 mm, 5 μm; Thermo Scientific) and an analytical column (PepMap100 C18, 50 cm × 75 μm i.d., 3 μm; Thermo Scientific). Mobile phase A was 0.1% (v/v) formic acid in LC-MS grade water; mobile phase B was 0.1% (v/v) formic acid in LC-MS grade acetonitrile. The peptides were resuspended in 20 μL mobile phase A, and first loaded onto a trap column at 6 μL/min, followed by separation on

an analytical column flowing at 250 nL/min. A linear LC gradient was applied from 1% to 25% mobile phase B over 125 min, followed by an increase to 32% mobile phase B over 10 min. The column was washed with 95% mobile phase B for 5 min, followed by equilibration with mobile phase A for 15 min. The spectra were collected on an Orbitrap Eclipse system installed with field asymmetric ion mobility spectrometry (FAIMS) Pro Interface. For the ion source settings, the spray voltage was set to 1.8 kV, funnel RF level at 30%, and heated capillary temperature at 275°C. The MS data were acquired in Orbitrap at 60,000 resolution, followed by MS/MS acquisition of the most intense precursors for 1 s. The MS1 scan range was set to 375–1600 m/z, AGC target was set to Standard, and the maximum injection time mode was set to Auto. For MS2 analysis, precursors with charge states 2–5 were selected. The isolation mode was Quadrupole, collision was by HCD at 30% normalized collision energy (NCE). The Orbitrap was set to detect MS2 fragments at 15,000 resolution with standard automatic gain control (AGC) target, 50 ms maximum injection time, and a 1.6 m/z isolation window. Dynamic exclusion was set to 30 s. Monoisotopic precursor selection (MIPS) was set to Peptide. For FAIMS settings, a 3-CV experiment (−40|-55|-75) was applied.

Proteome identification and quantitation

The MS raw files were processed using MaxQuant and Andromeda software suite (version 1.6.3.4).[75] Protein databases for human, mouse, E.coli and yeast were downloaded from UniProtKB website (https://www.uniprot.org/). The enzyme specificity was set to 'Trypsin'; variable modifications include oxidation of methionine, and acetyl (protein N-terminus); fixed modification includes carbamidomethylation of cysteine. The maximum missed cleavage sites were set to 2 and the minimum number of amino acids required for peptide identification was 7. The false discovery rate (FDR) was set to 1% for protein and peptide identifications. MaxLFQ function embedded in MaxQuant was enabled for label-free quantitation, and the LFQ minimum ratio count set to 1. Proteins identified as reverse hits, potential contaminants, or only by site-modification were filtered out from the "proteinGroups.txt" output file.

Experiment of studying RNA-binding proteins

Cell lines and cloning

Chemicals and reagents in this experiment were purchased from Millipore Sigma (St. Louis, MO) unless otherwise indicated. DLD-1 and HEK-293T cells were cultured in DMEM supplemented with 10% FBS and 1% penicillin/streptomycin. The plasmid carrying dCasRx-Basu was purchased from Addgene (Addgene, Watertown, MA, USA, Plasmid #153209) and guide RNA specific to the 3′UTR of linear NFIX or the back-splice junction of circNFIX were cloned into plasmid #138150 from Addgene. These vectors were assembled into lentiviruses by transfection into HEK-293T cells. Viral particles were collected 48 h after transfection and concentrated with lenti-X concentrator (Takara Bio, Kusatsu, Shiga, Japan, 631231) following manufacturer's protocol. DLD-1 cells were first transduced with dCasRx-BASU viruses and were allowed to grow for 48 h before cells are sorted for GFP using FACSAria Fusion High-Speed Cell Sorter (BD Biosciences, Franklin Lakes, NJ, USA). GFP+ sorted cells were then transduced with guide RNA viruses. Cells were allowed to grow for 48 h before being subjected to selection with puromycin (5 μg/mL) (Gibco, Waltham, MA).

CARPID

DLD-1 cells expressing both the dCasRx-BASU and specific guide RNA were incubated with 200 μM biotin for 15 min, washed with Phosphate-buffered saline (PBS) and then lysed in RIPA buffer supplemented with protease inhibitor. Cells were kept on ice for 30 min with occasional shaking and then centrifuged at 16,000 g for 15 min at 4°c. Biotinylated proteins were captured by incubating the cell lysates with Dynabeads MyOne Streptavidin C1 beads (Thermo Fisher) for 3 h at 4°C with rotation. Beads were then washed three times with 1.0 mL ice-cold lysis buffer followed by E3tip digestion as described above.

CircFISH and immunofluorescence

CircFISH was performed as described previously.[42] Briefly, DLD-1 cells were grown on coverslips and fixed with 4% formaldehyde and kept at 4°C in 70% ethanol. Coverslips were washed with a wash buffer and were then treated with Sodium borohydride washed three times for 15 min each. Cells were then blocked in 3 mg/mL bovine serum albumin (BSA) containing 2x saline sodium citrate solution for 30 min and then hybridized with YBX1 (Cell Signaling Technology, Danvers, MA) overnight at 4°c. Cells washed three times for 15 min each and blocked again for 30 min and hybridized with secondary antibody for 1 h at room temperature and then washed three times for 15 min each. Then cells were hybridized with probes in hybridization buffer and incubated overnight at 37°C water bath. Next, the coverslips were washed three times for 15 min each, stained with DAPI, and mounted in mounting media.

Fluorescence imaging and analysis

Images were captured with a 100× oil objective using a Nikon TiE Inverted epi fluorescence microscope equipped with a PIXIS 1024B camera (Princeton Instruments, Princeton, NJ). The images were obtained using Metamorph imaging software, version 7.8.13.0 (Molecular Devices, MA). z stack images were captured for each wavelength channel using 1.5-s exposures, for a total of 16 stacks, 0.2 μm apart. The compiled z stack images were analyzed using in-house designed algorithm with MATLAB software (MathWorks, Natick, MA) that identifies signals in each image and determines their three-dimensional coordinates, then identifies

spots that have a counterpart within a 250 nm distance in the other channel as described previously in detail.[74] The error bars indicate a 95% confidence interval. The *p*-values were obtained using Student's t test.

QUANTIFICATION AND STATISTICAL ANALYSIS

All the statistical analyses, including histograms, Pearson correlation, heatmap, volcano plot, and t-tests, were performed using either Perseus (version 1.6.2.3) or GraphPad Prism (version 9.5.1) if not indicated. For differential analysis, the LFQ values were log2 transformed, filtered by at least two valid values out of three replicates, and imputed using the default "normal distribution" method (width = 0.3, downshift = 1.8) in Perseus software. Significance was considered by Permutation FDR cutoff 0.05 and/or 0.01.

Cell Reports Methods, Volume *4*

Supplemental information

Development of an efficient, effective, and economical technology for proteome analysis

Katherine R. Martin, Ha T. Le, Ahmed Abdelgawad, Canyuan Yang, Guotao Lu, Jessica L. Keffer, Xiaohui Zhang, Zhihao Zhuang, Papa Nii Asare-Okai, Clara S. Chan, Mona Batish, and Yanbao Yu

Figure S1. Evaluation of glass bead membrane for proteomics analysis, related to Figure 1.

(A) Representative scanning electron microscopy imaging of the glass bead membrane (30 μm, 100X). (B) Comparison of protein and peptide identifications derived from the two types of GB membranes (9-13 μm, and 30 μm). E. coli samples were tested for this experiment. (C) Comparison of number of quantified proteins in different replicates. (D) Overlapping analyses of protein (upper panel) and peptide (lower panel) hits obtained by triplicate experiments of the three methods, E3filter, FASP, and SP4-GB.

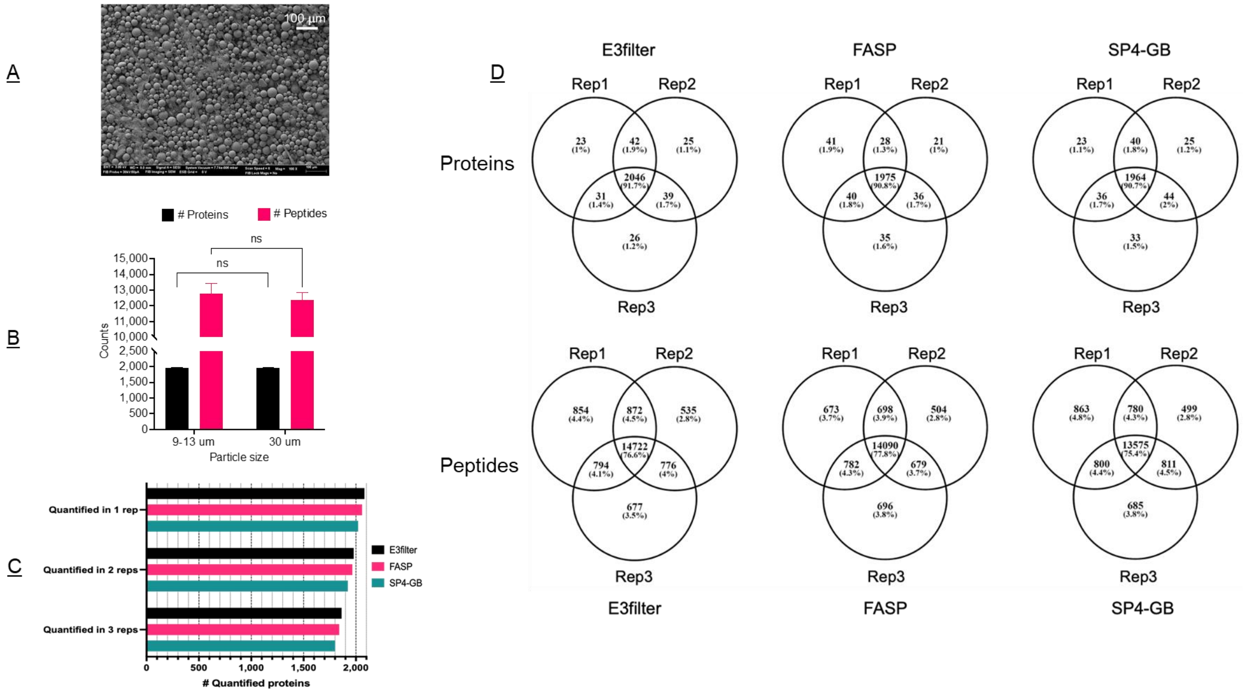

Figure S2. Proteomic comparison of E3tip and FA-SPEED methods, related to Figure 2.

(A) Correlation analysis of triplicate experiments of E3tip and FA-SPEED. Protein (left panel) and peptide (right panel) intensity (LFQ value, log2) of each experiment was plotted. Pearson r values (in blue) are depicted in the plots. (B) Comparison of FA-SPEED procedures with and without neutralization. Overlaps of *E.coli* protein and peptide identifications by the two procedures. The data were from three biological replicates. (C) Unsupervised hierarchical clustering analysis of the two procedures. Color bar indicates the protein intensity (LFQ, log2).

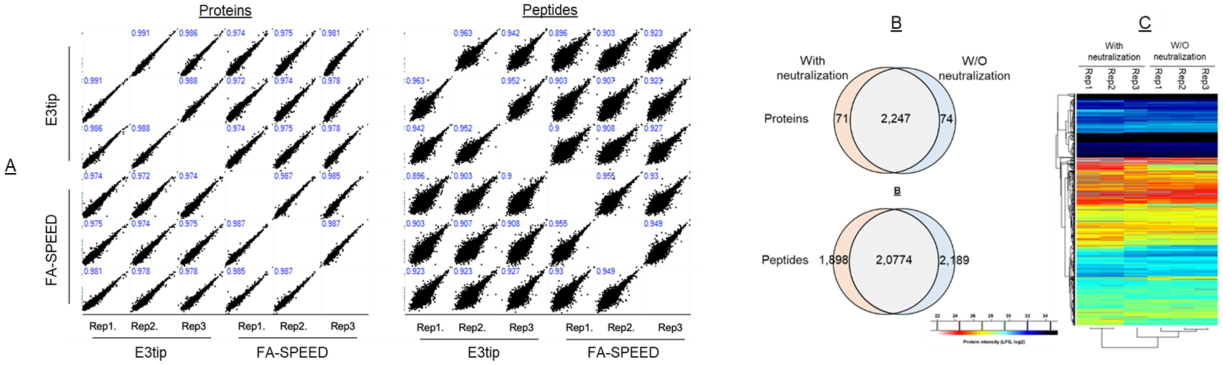

Figure S3. Applying E3tip to AP-MS analysis, related to Figure 5.

(A-B) Quantitative analyses of the two pulldown assays using linearRNA (A) and circRNA (B), respectively. The protein YBX1 was highlighted (red circle) in the plots. The curves indicate Permutation FDR 0.05. (C-H) Extended images of CircNFIX and YBX1 interaction. (C) DIC; (D) DAPI staining; (E) Raw merged z-stacks of Texas red (for probe set exclusively binding to linear RNA); (F) Raw merged z-stacks of Cy5 (probe set binding to exon found in both linear and circular RNA); (G) Raw merged z-stacks of Alexa 488 (anti-YBX1 antibody); (H) Merged image of the three channels showing colocalization. Full-length linear RNAs are represented as yellow, circular RNA as green, and YBX1 protein is represented in blue.

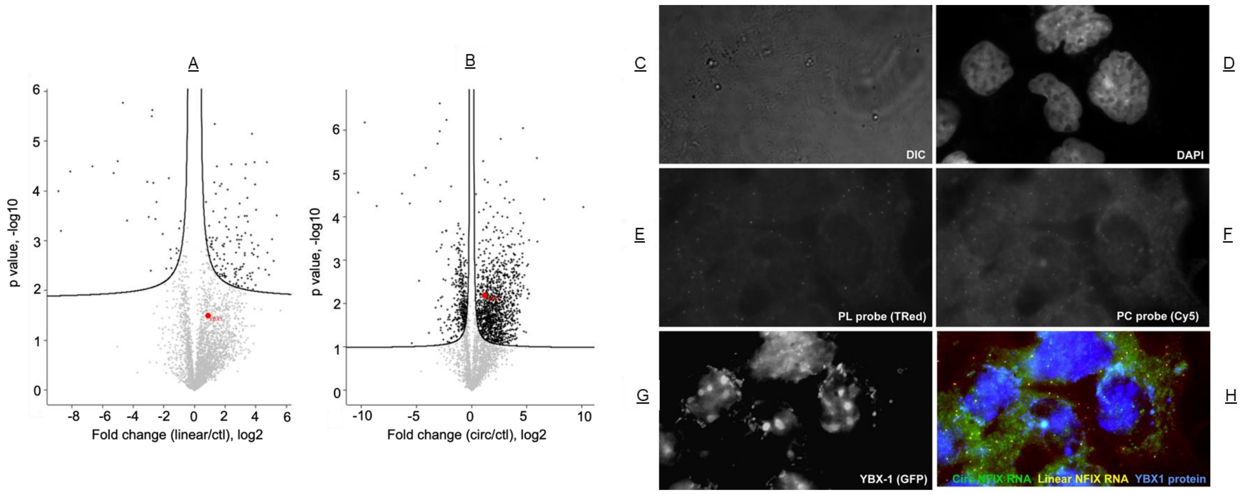

Figure S4. Evaluation of on-filter in-cell digestion, related to Figure 6.

(A) LCMS base peak profile of two representative digestions. Upper panel, on-filter in-cell digestion of ES-1 cells. Lower panel, SDS lysis of ES-1 cells and PES membrane followed by STrap digestion. (B-D) Proteomic comparison of “OFIC-E4” and “TFA-E3” digestion methods using *Leptothrix cholodnii* (SP-6) cells. The numbers of protein and peptide identifications, and the percentages of mis-cleavages were plotted. In this experiment, the SP6 cell pellets were either lysed with TFA followed by E3filter digestion (post protein precipitation), or on-filter in-cell digested with E4filter. Four biological replicates were included for each digestion experiment.

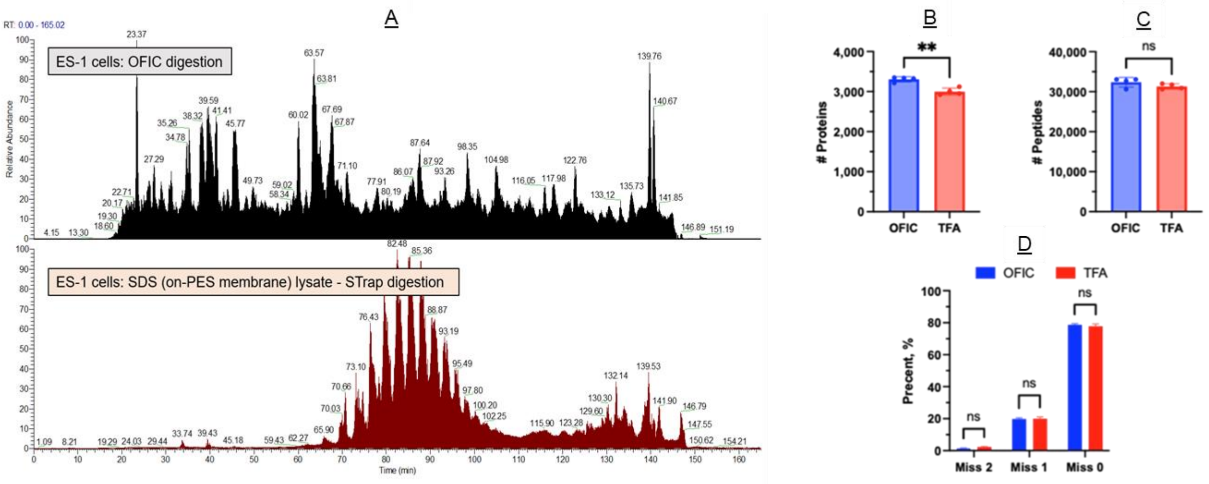

Table S1. Sequences of the probes used in this study, related to Figure 5.

probe name	Sequence of the probes	probe name	Sequence of the probes
PC probe set	tcgatgaacgggtggaactc	PL probe set	tcttatcagaggaaccagga
	Gcggacgtgaggcagcagtg		caagggctctctgagaactg
	Tgaaccaggtgtaggagaaa		tacttgacagagtccatgtc
	Ttgcgcttccgcgcctgcag		agaattgtgctggttgcttt
	Cttttcatgcttcttgaagt		attctgggacacgcaactag
	Gctcctcgtccttcgacatc		tcgagagcattccgactttt
	Cagcagctcgtccttcaccg		aactgaaagtcggcgagcag
	Gcttgatctcgggcttctcg		aaagtcgggggcacagaaa
	Agcagccgggatgcccactt		ccacccctaaataaagagta
	Gatgtccttgcgcagcttgg		tcccattccaacagcaaaag
	Agtcctcgcggaactcgggc		gttgctcaaatggaggagga
	Ttgcccgtgatggtcagcac		gtgtgttaccaaattgttcc
	Tgcccttctggtcggggttg		acaaggactcaagaaggggc
	Aggcagtcaatccgccggat		ttctctcagaggatcctgac
	Ccacaccttgtcagcctggc		ctcacaacaccacttgggaa
	Tcaccatgaccaggtccagc		aactctctgatgcattgcac
	Agggggatccccttaaacaa		cctcgtcaacgcaggaaaac
	Ccgctccccatcagtacttt		tcccaccgaaacagaacgaa
	Agcactgaggcgacttgtag		taatgtaagaagcaccaggg
	Tggacgcacaggccggggtt		tgcgtattcctaacaagtgc
	Tgtgactccaatgtgatgtg		ccgttccgttaaactcaaca
	Gataaagatccagttctttg		gtttgttcgttggcattgac
	Ggagtgtggacaaagtaagc		tggcgtctggctcaaagaag
			tgataatgctggtgagggtc
			ggggaggaaactaccaactt
			tcggggataggatgagagat
			aaatcgacctgtcagcgtgg
			tgtataaggcagtcgacagg
			gggtgagaacaaggcactag
			gggacgaaagttctcgtgac
			aacttgtttgacgggacggg
			ctcgtatatactgcgtttct
			agcacaccaaatccattagt
			tgtatctcagtgcagagacg
			agtaaactacccttgtttct

Supplemental Methods S1. Detailed protocols of E3technology and E4technolog, related to STAR Methods.

E3technology for proteomics sample preparation

Protein Digestion of Cell Lysate

Before starting

- Collect samples such as cell pellets, tissues, body fluids, etc.
- Lysis samples with buffers on your own choice.
- Calculate protein concentration and aliquot certain amount for proteomics.
- Estimated loading capacity: **E3tip**, <20 μg; **E3filter**, 10-100 μg; **E3cartridge**, 50-500 μg; **E3plate**, 20-200 μg.
- Ready-to-go E3 products are available.

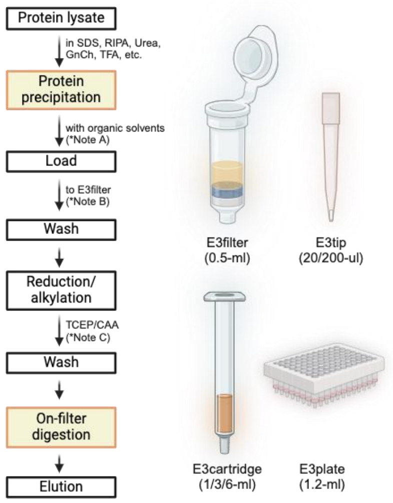

E3filter procedure

(Below is a representative procedure for E3filter. Please adjust buffer volume and centrifugation speed according to your filter type)

1. **Protein precipitation**
   Depending on sample volume, add 4x of 80% acetonitrile (ACN) to protein lysate to induce protein precipitation. **Note A**: For TFA lysate, acetone is recommended; for Guanidine hydrochloride lysate, ethanol is recommended for protein precipitation.
2. **Sample loading**
   Transfer protein precipitate to E3filters, centrifuge at 400 x g for 1-2 min; discard flow through. Centrifugation speed may be increased (up to 7,000 x g) if more proteins are loaded. **Note B**: when handling low-input samples (e.g., <10 μg), the protein lysate may be added directly to E3filter followed by protein precipitation with organics.
3. **Wash**
   Add 200 μl 80% ACN, centrifuge at 400 x g for 1-2 min, and discard flow through. Repeat this step 2-3 times.
4. **Reduction and alkylation**
   Add 100 μl 50 mM triethylammonium bicarbonate (TEAB), 10 mM Tris(2-carboxyethyl)phosphine (TCEP), and 40mM chloroacetamide (CAA), incubate at 45°C for 5 min with gentle shaking. Spin and discard flow through.
   Note C: this step may be skipped if it were performed ahead (e.g., during cell lysis step).
5. **Wash**
   Same as Step 3.
6. **Digestion**
   Transfer E3filters to clean collection tubes, add 100-200 μl 50 mM TEAB, and desired enzyme (Trypsin or Trypsin/Lys-C mix) at 1:50 ratio. Incubate E3filters at 37°C for 16-18 hours with gentle shaking (e.g., 300-500 rpm).
7. **Elution**
   Centrifuge E3filters at 400 x g for 1-2 min, transfer elutes to new collection tubes. Perform two additional elution steps with 0.1% formic acid in water, and 50% acetonitrile/0.1% formic acid in water, respectively. Pool the elution, dry, and proceed to desalting, or store at -80°C until further use.

Martin et al., Development of an efficient, effective, and economical technology for proteome analysis, Cell Reports Methods (2024), https://doi.org/10.1016/j.crmeth.2024.100796

E4technology for proteomic sample preparation

Protein Digestion of Intact Cells

Before starting

- Collect fresh cell pellets or tissue slices, rinse with cold PBS to remove excess culturing media or blood.
- Count cells and aliquot certain amount for proteomics.
- Estimated capacity: **E4tip**, ≤ 50,000 cells; **E4filter**, 10,000-100,000 cells; **E4cartridge**, 100,000-1,000,000 cells; **E4plate**, 50,000-500,000 cells.
- Ready-to-go E4 products are available.

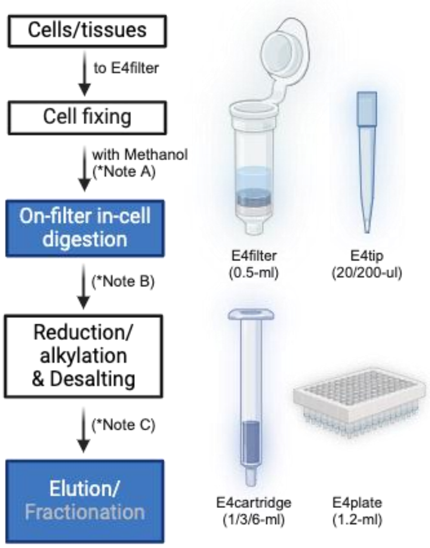

E4 procedure

(Below is a representative procedure for E4filter; please adjust buffer volume and centrifugation speed according to your filter type)

1. **Cell loading and fixing**
   Transfer certain amount of cells to E4filter, add 200 μl of 100% methanol, and mix cells by gentle pipetting. Incubate on ice or at 4°C for 0.5-2.0 hours. Centrifuge at 1,500 x g for 1-2 min, and discard flow through. Here, centrifugation speed may go up to 7,000 x g if more cells are loaded.
   Note A: The flow through (extraction) here may be collected for ***metabolomics*** analysis.
2. **Digestion**
   Transfer E4filters to clean collection tubes, add 100-200 μl 50 mM TEAB, and desired enzyme (Trypsin or Trypsin/Lys-C mix) at 1:50 ratio. Incubate at 37°C for 16-18 hours with gentle shaking (e.g., 300-500 rpm). For E4tips, no caps are required during incubation. Only minor liquid loss from evaporation can be seen.
3. **Reduction/alkylation**
   After digestion, before doing any centrifugation, add 10 mM (final concentration) Tris(2-carboxyethyl)phosphine (TCEP) and 40mM (final concentration) chloroacetamide (CAA), incubate at 45°C for 5 min with gentle shaking.
4. **Acidification and desalting**
   Add formic acid to final concentration of 1%, centrifuge at 1,500 x g for 1-2 min, and discard flow through. Add 200 μl 0.5% acetic acid in water, spin and discard flow through. The desalting step may be repeated one more time.
   Note B: If E4tips are used, the tips are now desalted, and are ready for elution (**Step 5**), in-tip high-pH fractionation, or direct LCMS acquisition (e.g., if EvoSep LC is accessible).
5. **Elution (or Fractionation)**
   Transfer E4filters to clean collection tubes. Do two sequential elution by adding 200 μl 60% acetonitrile/0.5% acetic acid in water (elution I), and 80% acetonitrile/0.5% acetic acid in water (elution II), respectively. Centrifuge at 1,500 x g for 1-2 min, pool the elution, dry, store at -80°C until LCMS analysis.
   Note C: If in-depth proteome coverage is desired, high pH fractionation may be carried out at this step (by doing sequential elution).

Martin et al., Development of an efficient, effective, and economical technology for proteome analysis,
Cell Reports Methods (2024), https://doi.org/10.1016/j.crmeth.2024.100796
